# *Reln* mRNA Delivery Drives Neurogenic Bone Regeneration by Orchestrating Schwann Cell‐Mediated Exosomal Trafficking

**DOI:** 10.1002/advs.78530

**Published:** 2026-11-02

**Authors:** Shuai Lin, Qianwen Li, Duo Liu, Mingzhao Li, Han Zhang, Liujing Chen, Yan Yu, Jiuhui Jiang, Chun Xu, Ruili Yang

**Affiliations:** ^1^ Department of Orthodontics Peking University School and Hospital of Stomatology National Center for Stomatology & National Clinical Research Center for Oral Diseases & National Engineering Research Center of Oral Biomaterials and Digital Medical Devices Beijing Key Laboratory of Digital Stomatology NHC Key Laboratory of Digital Stomatology NMPA Key Laboratory for Dental Materials Beijing China; ^2^ Sydney Dental School Faculty of Medicine and Health The University of Sydney Camperdown Australia; ^3^ Charles Perkins Centre The University of Sydney Camperdown Australia; ^4^ School of Stomatology Sanya Division (Sanya Stomatology Center) Peking University Sanya Hainan China

**Keywords:** exosomal endocytosis, mRNA delivery, neurogenic bone regeneration, Reelin, Schwann cells

## Abstract

Neurogenic bone regeneration is essential for the effective restoration of bone tissue functionality, while the mechanisms and new strategies underlying neurogenic bone regeneration remain to be further explored. Here, single‐cell transcriptomic analysis revealed enhanced mesenchymal‐Schwann cell crosstalk during bone regeneration. Functionally, we verify that Schwann cells are indispensable for bone regeneration via paracrine exosome‐mediated crosstalk with MSCs, a process promoted by Reelin. Mechanistically, Reelin enhances Schwann cell‐derived exosome internalization by MSCs via regulating exosomal proteins, especially UBE2K, which promotes MSC osteogenic differentiation through the ERK pathway, associated with Lys63‐linked polyubiquitination. For effective neurogenic bone regeneration, we engineered mesoporous silica nanoparticles (MSNs) as a CRISPRa delivery system for local *Reln* gene activation at the defect area. This mRNA delivery system could promote bone regeneration in both calvarial defect and tibial fracture models by constructing a pro‐osteogenic neurogenic microenvironment. Collectively, our findings uncover a Reelin‐Schwann cell exosomes‐ERK regulatory axis in bone regeneration with translational potential, highlighting the therapeutic value of targeting neural‐osteogenic crosstalk in bone regeneration.

## Graphical abstract

MSNs serve as a robust vehicle for CRISPRa‐mediated *Reln* induction, driving bone repair through the establishment of a neurogenic‐supportive microenvironment.

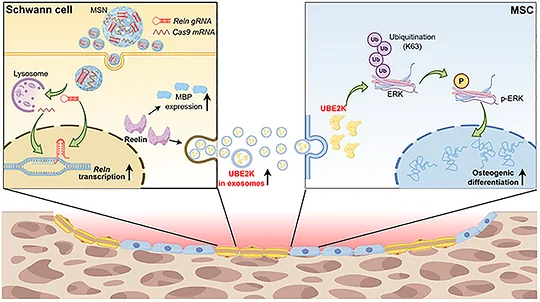

## 1 Introduction

Bone defect repair represents a well‐orchestrated biological process governed by the local bone microenvironment, which coordinates osteogenic differentiation of bone marrow mesenchymal stem cell (MSC) and osteoclast‐mediated bone resorption [1]. Beyond canonical cellular and humoral regulators, accumulating evidence highlights peripheral nerves as indispensable players in bone regeneration. Nerve fibers are widely distributed throughout cortical bone, cancellous bone, bone marrow, and periosteum [2], with higher densities observed in metabolically active regions such as the metaphysis [3]. This extensive innervation establishes a crucial physiological foundation for the regulation of bone regeneration by peripheral nerves. Moreover, bone metabolism is modulated through the local release of neurotransmitters, neuropeptides, and neurotrophic factors, which interact with bone cells and matrix [4, 5, 6, 7]. However, the underlying mechanisms by which peripheral nerves regulate bone repair remain to be elucidated.

Schwann cells, the myelinating glia of the peripheral nervous system, are reported to support neuronal function by ensheathing axons to form myelin [8]. Moreover, Schwann cells have been implicated in tissue repair processes, including digit tip regeneration in mice [9], skin wound healing [10], periodontal tissue regeneration [11], and dental pulp regeneration [12]. Importantly, Schwann cell‐derived factors such as PDGF‐AA, OSM, and PTH have been reported to be essential for the repair of mandibular defects by skeletal stem cells [13]. In periodontal tissue repair, Schwann cells secrete brain‐derived neurotrophic factor to support the maturation and mineralization of alveolar bone [14]. More recently, Schwann cell‐derived exosomes functionalized 3D‐printed titanium scaffolds facilitate bone ingrowth and enhance the osseointegration of metallic implants [15]. However, precisely regulating the crosstalk between Schwann cells and MSCs to enhance bone regeneration remains a challenge.

To effectively modulate the cellular interactions underlying neurogenic bone regeneration, precise control over gene expression within the injury microenvironment is required. RNA‐based therapeutics have emerged as a promising strategy for such regulation, enabling transient and programmable control of cellular behavior. Various RNA molecules, including mRNA, siRNA, miRNA, and gRNA, have been explored for their ability to regulate osteogenic differentiation and bone regeneration [16]. In particular, the co‐delivery of *Cas9* mRNA and gRNA, which facilitates site‐specific gene activation, offers a versatile strategy for modulating endogenous protein expression with high precision [17, 18]. However, the therapeutic potential of these approaches is critically dependent on the development of robust delivery systems. Functional biomaterials facilitate bone regeneration by providing bioelectric stimulation [19] or enabling targeted cargo delivery [20]. Conventional lipid‐based nanoparticles, while widely used, suffer from structural instability and reduced transfection efficiency under acidic and inflammatory conditions [21]. Mesoporous silica nanoparticles (MSNs) offer a promising alternative due to their well‐defined porous architecture, high surface area, and superior physicochemical stability [22, 23, 24, 25], which enable efficient loading and protection of nucleic acid cargos. Despite these advantages, the utilization of MSNs to implement the co‐delivery of *Cas9* mRNA and gRNA strategy for targeted endogenous protein regulation remains largely unexplored.

In this study, we identified that Reelin promoted neurogenic bone regeneration by enhancing the endocytosis of Schwann cell‐derived exosomes, which activated ERK via UBE2K‐mediated K63 ubiquitination. For high‐efficiency and cost‐effective translational applications, we designed an MSN‐based delivery system (MSN‐CRISPRa_*Reln*) to deliver the *Reln* gRNA and *Cas9* mRNA into Schwann cells, triggering robust Reelin expression and significantly enhancing bone regeneration by establishing a pro‐osteogenic neurogenic microenvironment. These findings highlight the therapeutic potential of MSN‐mediated local mRNA delivery as an efficient platform for bone regeneration.

## 2 Results

### 2.1 Crosstalk Between Schwann Cells and Mesenchymal Cells Plays a Key Role in Bone Regeneration

To figure out the crucial regulatory factors on bone regeneration, a published single‐cell dataset of mouse calvarial bone was analyzed to elucidate its cellular and molecular basis. Cells from uninjured (7631 cells) and injured (7 days post‐injury, 6678 cells) calvarial bone were classified into 12 major clusters based on their transcriptional profiles (Figure S1A, Figure 1A). Compared with the uninjured group, the number of predicted cell–cell interactions was markedly increased in the 7 days post‐injury group (Figure 1B,C, Figure S1B). Differential network analysis revealed that the mesenchymal‐Schwann cell crosstalk represented one of the most significantly altered interaction pairs, suggesting a potentially critical partnership in orchestrating bone regeneration (Figure S1B,C). To further investigate the role of Schwann cells in bone regeneration, *Plp‐Cre^ERT2^*; *Ai14* mice were generated [26].

**FIGURE 1:**
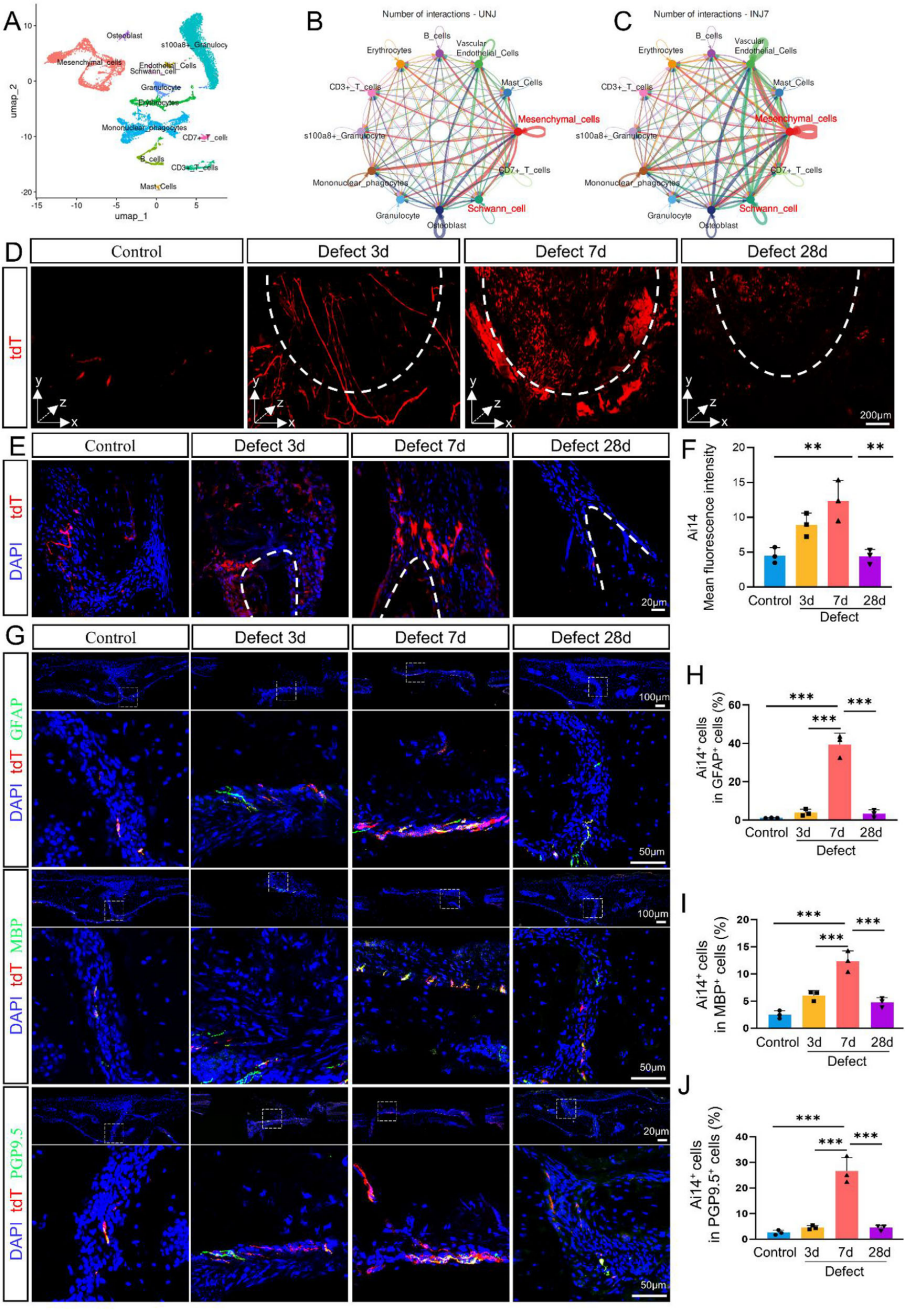
Crosstalk between Schwann cells and mesenchymal cells plays a key role in bone regeneration. (A) UMAP plot showed cell cluster results of mouse calvarial bone before and after injury. (B‐C) Cell–cell interaction network diagrams depicted the number of interactions in uninjured (B, UNJ) and injured (C, INJ7) conditions. (D‐E) The Schwann cells labeled by tdTomato in injury boundary using *Plp‐Cre^ERT2^; Ai14* mice, as presented by (D) projection of 3D images after TESOS clearing and (E) frozen sections at 0‐, 3‐, 7‐, and 28‐days post‐injury. (F) Quantitative analysis of tdTomato positive Schwann cells density at 0‐, 3‐, 7‐, and 28‐days post‐injury. (G) The expression of GFAP (upper panel), MBP (middle panel), and PGP9.5 (lower panel) in the calvarial defect area at 0‐, 3‐, 7‐, and 28‐days post‐injury, presented by immunofluorescence staining from *Plp‐Cre^ERT2^; Ai14* mice. (H‐J) Quantification of immunofluorescence intensity for GFAP (H), MBP (I), and PGP9.5 (J) at 0‐, 3‐, 7‐, and 28‐days post‐injury. (**p < 0.01, ***p < 0.001). Each dot represents an individual replicate (*n* = 3 in F, H, I, J). Statistical significance was analyzed by One‐way ANOVA with Bonferroni post‐hoc test (F, H, I, J).

Following tamoxifen induction for 3 consecutive days at 6–8 weeks of age, tdTomato expression was shown in the bone marrow, periosteum, and skull (Figure S1D). Next, to track cellular dynamics during repair, mice were subjected to calvarial defect surgery 2 weeks after tamoxifen clearance, and harvested at 3, 7, and 28 days post‐injury (Figure S1E). Micro‐CT analysis showed progressive new bone formation extending from the defect margins over time (Figure S1F). H&E staining showed time‐dependent histological healing and bone matrix deposition (Figure S1G). These findings establish a structural baseline for evaluating Schwann cell dynamics during bone regeneration. 3D Tissue clearing deep imaging demonstrated that *Plp^+^* Schwann cells rapidly migrated toward the defect margins by day 3, peaked in number and clustered along the injury border at day 7, and subsequently decreased by day 28 (Figure 1D). This dynamic change of *Plp^+^* Schwann cells after bone defects was also verified by the immunofluorescence staining (Figure 1E,F). The immunostaining pattern on day 7 calvarial sections matches the endogenous tdTomato fluorescence signal, validating its specificity (Figure S1H). Moreover, the expression of neural lineage markers GFAP and MBP was markedly upregulated at the defect site after 7 days bone defects, accompanied by abundant PGP9.5‐positive nerve fibers (Figure 1G–J). These findings indicate that Schwann cells participate in the bone regeneration process via neural remodeling.

### 2.2 Schwann Cells Are Essential for Bone Regeneration via Paracrine Signaling

To determine whether Schwann cells are required for bone regeneration, the *DTA* mice were used to mate with *Plp‐Cre^ERT2^*; *Ai14* to specifically ablate *Plp^+^* Schwann cells. In *Plp‐Cre^ERT2^; Ai14* mice, injury markedly increased tdTomato signal at the defect site. In contrast, *Plp‐Cre^ERT2^; Ai14; DTA* mice showed an 81.51% ± 11.04% reduction in baseline tdTomato intensity, which remained suppressed after injury (Figure 2A,B), confirming efficient ablation of *Plp* ^+^ Schwann cells (Figure 2A,B). At 28 days post‐injury, bone morphology in uninjured calvaria were comparable between control and DTA group (Figure 2C–E). However, Schwann cell ablation led to decreased bone regeneration after the injury, accompanied by significant disruption of bone microarchitecture (Figure 2C–E). Histologically, there were less bone volume and more disorganized bone structure in DTA mice compared with control ones (Figure 2F). Moreover, the expression of RUNX2 in the DTA group was lower than that in the control group (Figure 2G,H). These results imply that the Schwann cells are indispensable for effective bone regeneration.

**FIGURE 2:**
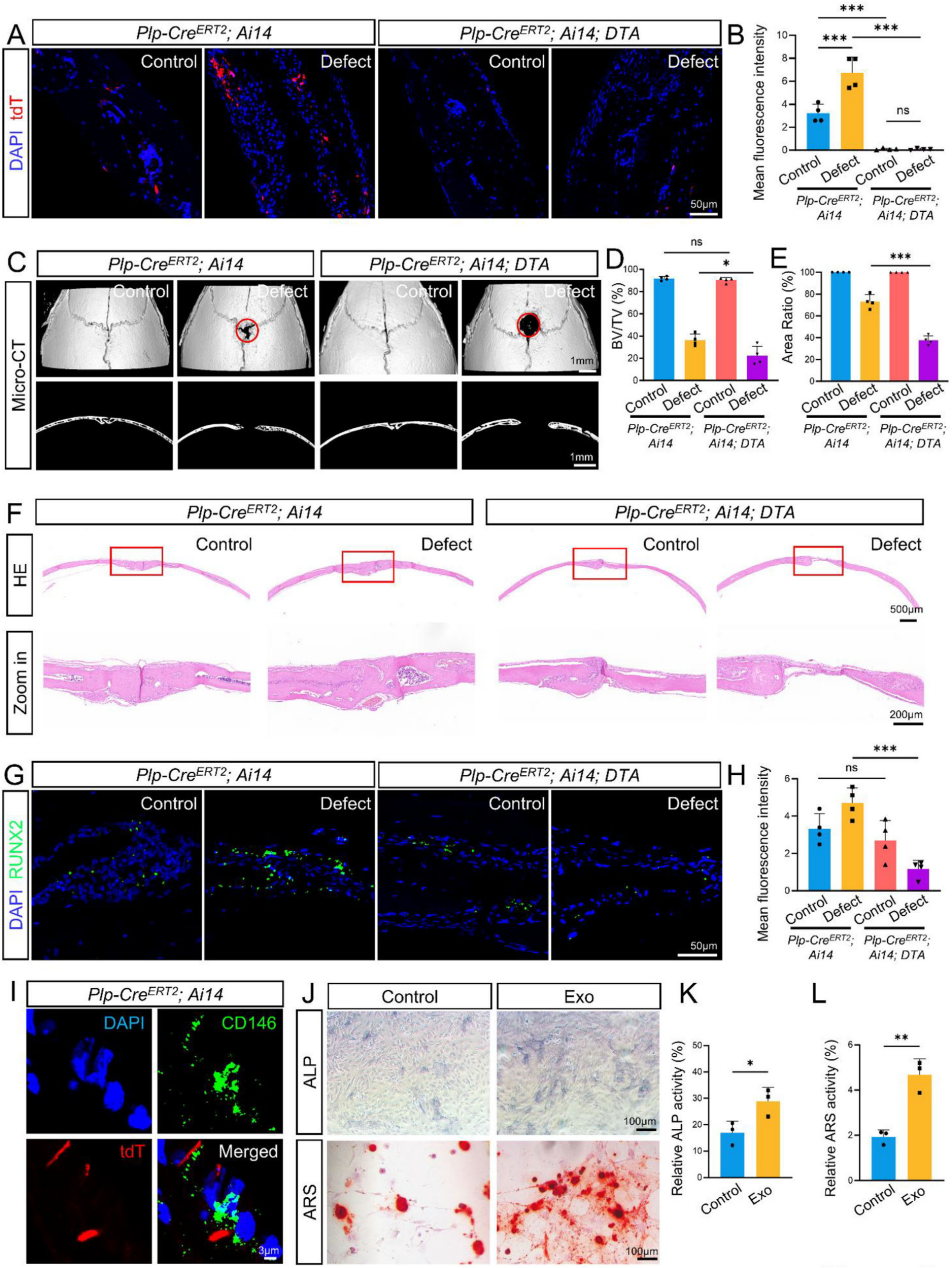
Schwann cells are required for calvarial bone regeneration via paracrine exosomes. (A) Representative images showing tdTomato‐labeled cells in the calvarial defect area of *Plp‐Cre^ERT2^; Ai14* and *Plp‐Cre^ERT2^; Ai14; DTA* mice at 4 weeks post‐injury. (B) Quantitative analysis of tdTomato positive Schwann cells density in *Plp‐Cre^ERT2^; Ai14* and *Plp‐Cre^ERT2^; Ai14; DTA* mice with or without injury. (C) Representative 3D micro‐CT reconstructions and coronal 2D sections of the calvarial defects in *Plp‐Cre^ERT2^; Ai14* and *Plp‐Cre^ERT2^; Ai14; DTA* mice at 4 weeks post‐injury. (D‐E) Quantitative analysis of BV/TV and defect area ratio in regeneration area determined by micro‐CT measurements at 4 weeks post‐injury. (F) H&E staining of calvarial bone in *Plp‐Cre^ERT2^; Ai14* mice and *Plp‐Cre^ERT2^; Ai14; DTA* mice. (G) The expression of RUNX2 in calvarial bone with or without defects, presented by immunofluorescence staining from *Plp‐Cre^ERT2^; Ai14* mice and *Plp‐Cre^ERT2^; Ai14; DTA* mice. (H) Quantitative analysis of RUNX2 density shown in (G). (I) The expression of tdTomato and CD90 in calvarial bone, presented by immunofluorescence staining from *Plp‐Cre^ERT2^; Ai14* mice. (J) The ALP and ARS staining in control and Schwann cell–derived exosomes treated groups after osteogenic induction. (K, L) Quantification of ALP and ARS staining in (J). Data are presented by Mean ± SD. (*p < 0.05, **p < 0.01, ***p < 0.001). Each dot represents an individual repicate  (*n* = 3 in K, L; *n* = 4 in B, D, E, H). Statistical significance was analyzed by One‐way ANOVA with Bonferroni post‐hoc test (B, D, E, H). Statistical significance was analyzed by unpaired Student's *t* test (K, L).

As Schwann cells were found to be spatially adjacent to MSCs, and significant crosstalk between the two cell types was indicated by single cell sequencing analysis (Figures 1B and 2I), we assessed the effect of Schwann cell on the osteogenic differentiation of MSCs. In a scratch assay, MSCs treated with Schwann cell‐conditioned medium displayed markedly higher migration ability compared to control MSCs (Figure S2A,B). Furthermore, Schwann cell‐conditioned medium increased osteogenic differentiation of MSCs, shown as higher activity of ALP and Alizarin red staining (Figure S2C–E).

Exosomes, as key secretory vesicles, are reported to serve essential roles in diverse biological processes [27]. Next, we purified the exosomes from Schwann cells to treat MSCs. The results revealed that Schwann cell‐derived exosomes also promoted MSC osteogenic differentiation, shown by significantly enhancing activity of ALP and Alizarin red staining (Figure 2J–L). Collectively, these results demonstrate that Schwann cell‐derived exosomes act as functional mediators that promote MSC migration and osteogenic differentiation.

### 2.3 Reelin Promotes Osteogenesis of MSCs by Enhancing Schwann Cell‐Derived Exosomes Endocytosis

Our previous study showed that Reelin could promote tendon regeneration [28]. Given the crucial role of Reelin in brain development and adult neural protection [29, 30], we characterized the expression of Reelin at the bone defect site. Immunofluorescence staining showed that the Reelin fluorescence signal did not co‐localize with tdTomato labeled Schwann cells, suggesting a paracrine origin of Reelin in the defect microenvironment (Figure S3). We next analyzed the effects of Reelin in Schwann cell exosome‐mediated osteogenic differentiation. The results demonstrated that the osteogenic differentiation of MSCs was further enhanced by Reelin‐stimulated exosomes treatment compared to control exosomes, as shown by increased ALP activity and Alizarin red staining (Figure 3A–C).

**FIGURE 3:**
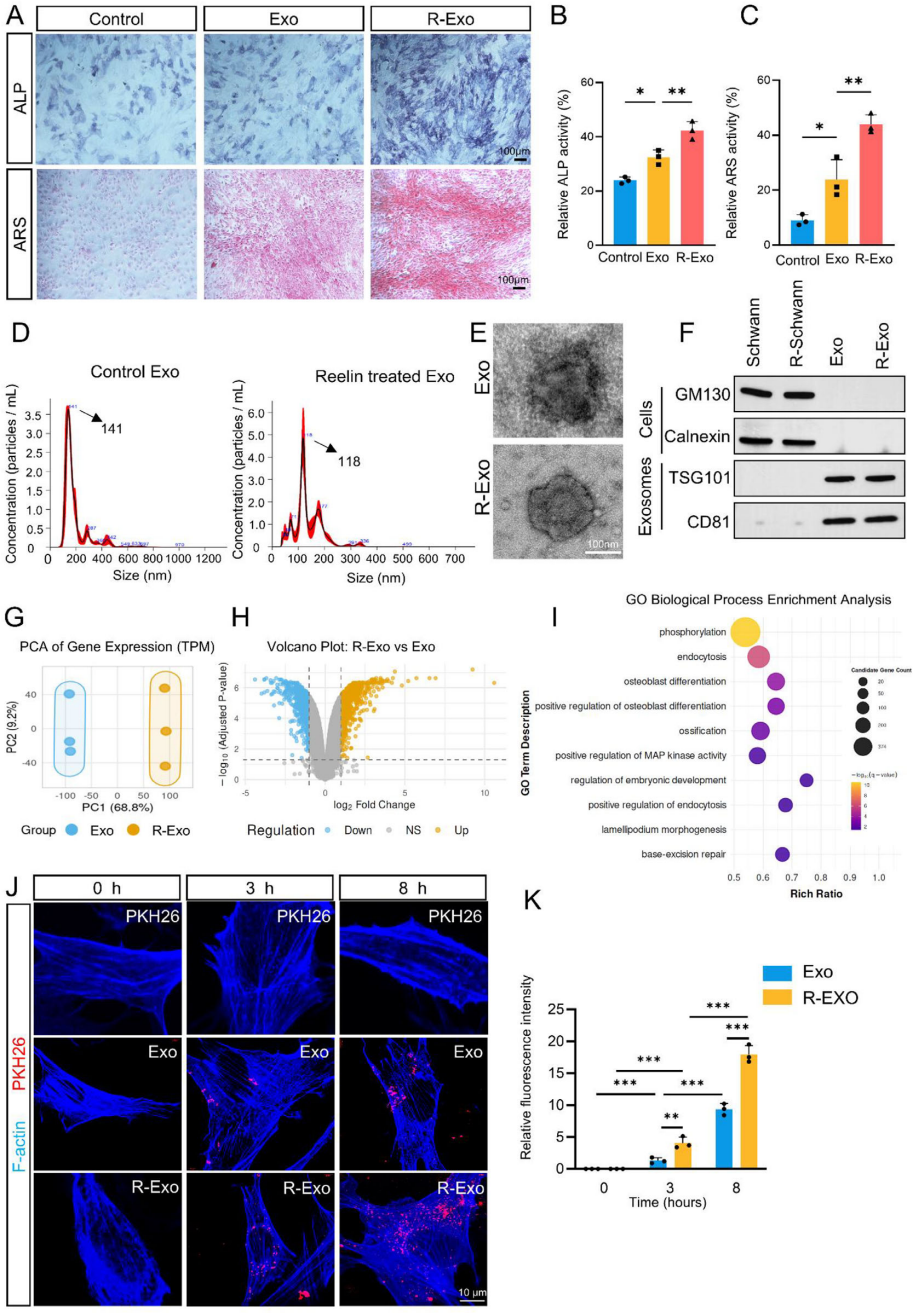
Reelin promotes osteogenesis of MSCs by enhancing Schwann cell‐derived exosomes endocytosis. (A) The ALP and ARS staining in control, exosomes, and Reelin‐stimulated exosomes treated groups after osteogenic induction. (B, C) Quantification of ALP and ARS staining in control, exosomes, and Reelin‐stimulated exosomes treated groups. (D) Nanoparticle tracking analysis (NTA) showed size distribution of control exosomes and Reelin‐stimulated exosomes. (E) Transmission electron microscopy image of isolated exosomes in exosomes and Reelin‐stimulated exosomes groups. (F) The expression of GM130, Calnexin, CD81, and TSG101 in Schwann cells, Reelin‐stimulated Schwann cells, exosomes, and Reelin‐stimulated exosomes groups, assessed by Western blotting. (G) PCA of the bulk RNA‐seq transcriptomes in MSCs treated with exosomes or Reelin‐stimulated exosomes. (H) Volcano plot of differentially expressed genes in MSCs treated with exosomes or Reelin‐stimulated exosomes (adjusted *p* < 0.05 and |log_2_FC| > 1). (I) Biological process enrichment in Reelin‐stimulated exosomes group assessed by GO analysis. (J, K) The immunofluorescence staining and quantitative results of exosome internalization in exosomes and Reelin‐stimulated exosomes groups after treatment for 0, 3, 8 h. Exo: Schwann cell–derived exosomes; R‐Exo: Reelin‐stimulated Schwann cell–derived exosomes. (*p < 0.05, **p < 0.01, ***p < 0.001). Each dot represents one individual replicate (*n* = 3 in B, C, K). Statistical significance was analyzed by One‐way ANOVA with Bonferroni post‐hoc test (B, C). Statistical significance was analyzed by Two‐way ANOVA with Bonferroni post‐hoc test (K).

Next, we explored how Reelin regulated Schwann cell‐derived exosomes to promote MSC osteogenic differentiation. We first analyzed whether Reelin treatment altered the synthesis and secretion of the exosomes. However, no differences in the quantity and diameter of control exosomes and Reelin‐stimulated exosomes were detected, as analyzed by Nanoparticle Tracking Analysis (NTA) and ‌Transmission Electron Microscope (TEM), respectively (Figure 3D,E). In addition, Reelin treatment failed to alter the levels of the exosomal positive markers CD81 and TSG101 in Schwann cell‐derived exosomes, as assessed by Western blot. Meanwhile, the negative markers GM130 and Calnexin were undetectable, verifying the purity of the isolated exosomal fractions (Figure 3F). Thus, the RNA sequencing analysis was employed to figure out the cellular response of MSCs treated with Reelin‐stimulated exosomes. A distinct transcriptomic separation between the two groups was demonstrated by principal component analysis (PCA) (Figure 3G). Furthermore, a total of 809 upregulated and 659 downregulated differentially expressed genes were identified in MSCs following treatment with Reelin‐stimulated exosomes (Figure 3H). The GO analysis indicated that the endocytic process related genes were enriched after Reelin treatment (Figure 3I).

To analyze the role of Reelin in exosome endocytosis, we labeled exosomes with PKH26 to treat MSCs. The results showed that the endocytic exosomes, shown by red fluorescence, was enriched in MSCs in a time‐dependent manner (Figure S4A,B). Moreover, significantly more Reelin‐stimulated exosomes were internalized by MSCs compared with control ones (Figure 3J,K). Together, these results identify Reelin as a key factor that facilitates bone regeneration by promoting the endocytosis of Schwann cell‐derived exosomes into MSCs.

### 2.4 Reelin‐Stimulated Exosomal UBE2K Promotes ERK Activation via Lys63‐Linked Polyubiquitination

Next, to explore the underlying mechanism for Reelin regulating exosome endocytosis, we further analyzed the different proteins between control and Reelin stimulated exosomes by mass spectrometry analysis. A comparable number of proteins was identified across all six samples (Figure 4A). The results showed that UBE2K, an E2 ubiquitin‐conjugating enzyme, was highly expressed in Reelin‐stimulated exosomes (Figure 4B). The Western blot analysis verified that Reelin treatment could increase the level of UBE2K in exosomes (Figure 4C). Next, to trace direct exosomal delivery, we incubated recipient MSCs with exosomes derived from UBE2K‐Myc‐overexpressing Schwann cells and successfully detected the Myc‐tag in MSCs, indicating donor‐to‐recipient transfer (Figure S5A). Functionally, exosomes derived from UBE2K‐overexpressing Schwann cells increased their endocytosis by MSCs (Figure 4D,E). Collectively, these data indicate that exosome‐associated UBE2K promotes the uptake of Reelin‐stimulated exosomes by recipient MSCs.

**FIGURE 4:**
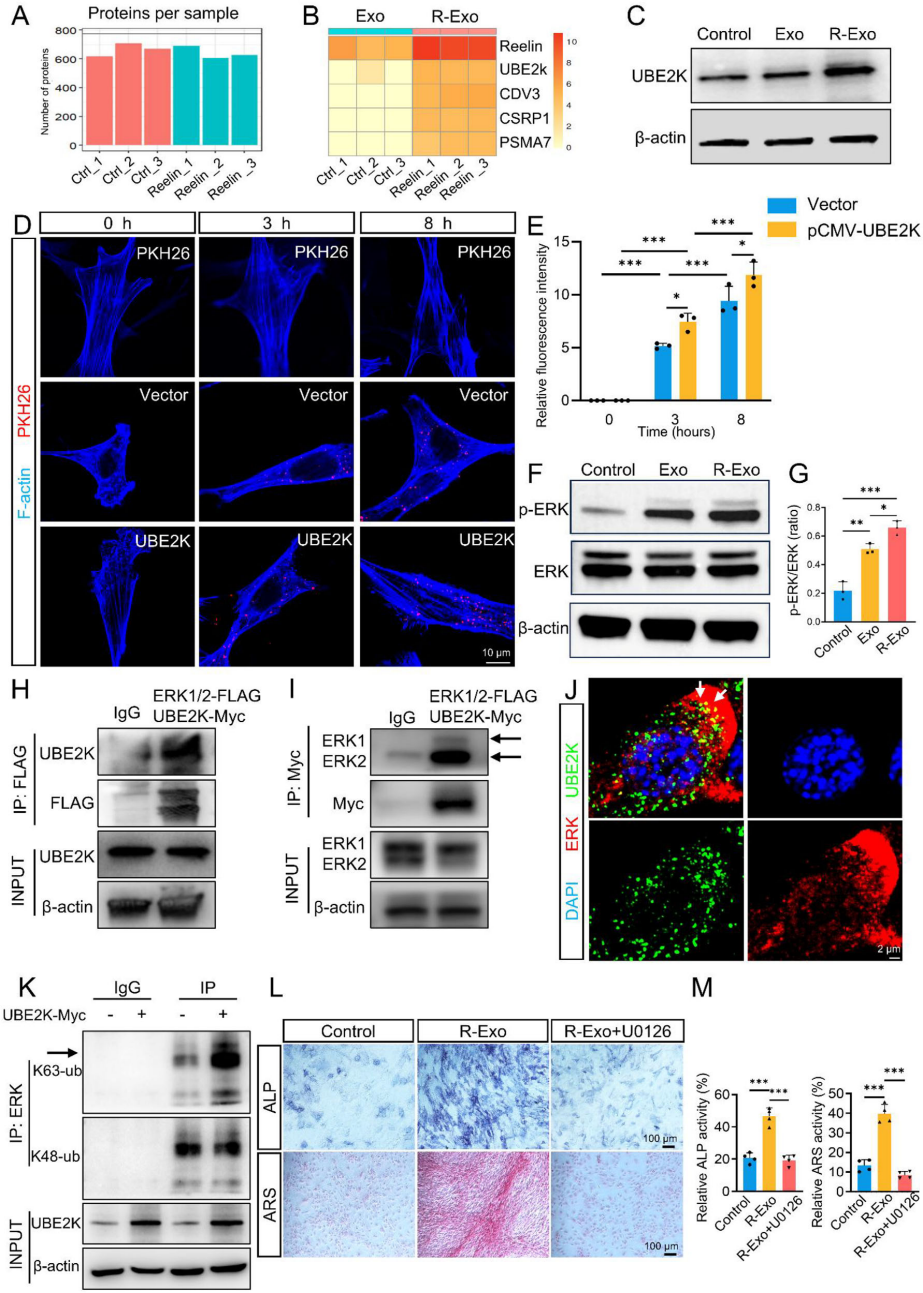
Reelin‐stimulated exosomal UBE2K promotes ERK activation via Lys63‐linked polyubiquitination. (A) The number of identified proteins in control and Reelin treated exosomes assessed by mass spectrometry. (B) The top 5 significantly increased proteins in Reelin treated exosomes based on the different proteins comparison between control and Reelin‐stimulated exosomes. (C) The expression of UBE2K in control, exosomes, and Reelin‐stimulated exosomes groups, assessed by Western blotting. (D, E) The immunofluorescence staining and quantitative results of exosome internalization in control and UBE2K overexpressed groups after treatment for 0, 3, and 8 h. (F, G) The expression and quantitative results of p‐ERK, ERK in control, exosomes, and Reelin‐stimulated exosomes groups, assessed by Western blotting. (H, I) Co‐IP evaluated the protein binding of UBE2K and ERK in Lenti‐X 293T cells, which were transfected with pCMV‐ERK1‐FLAG, pCMV‐ERK2‐FLAG, pCMV‐UBE2K‐Myc. (J) The expression of UBE2K (green) and ERK (red) in MSC, presented by immunofluorescence staining. (K) Co‐IP evaluated the protein level of ubiquitinated ERK in MSC, which were transfected with the Vector or pCMV‐UBE2K‐Myc plasmids. (L) The ALP and ARS staining in control and Reelin stimulated exosomes treated groups with or without U0126 treatment. (M) Quantification of ALP and ARS staining in (L). Exo: Schwann cell–derived exosomes; R‐Exo: Reelin‐stimulated Schwann cell–derived exosomes. (*p < 0.05, **p < 0.01, ***p < 0.001). Each dot represents one individual replicate (*n* = 3 in E, G; *n* = 4 in M). Statistical significance was analyzed by Two‐way ANOVA with Bonferroni post‐hoc test (E). Statistical significance was analyzed by One‐way ANOVA with Bonferroni post‐hoc test (G, M).

We next investigated whether delivered UBE2K also acts intracellularly in recipient cells. We compared the gene expression profiles between control exosome and Reelin stimulated exosome treated MSCs by RNA‐sequencing analysis. The results revealed that the endocytosis, TNF signaling, and MAPK signaling pathways were significantly increased (Figure S5B). Notably, UBE2K is an E2 ubiquitin‐conjugating enzyme, and ERK/MAPK signaling has been reported to be activated via Lys63‐linked ubiquitination [31]. Therefore, we hypothesized that exosome‐delivered UBE2K activates ERK via Lys63‐linked polyubiquitination. GSEA corroborated a prominent enrichment of the MAPK signaling in Reelin‐stimulated exosome‐treated MSCs (Figure S5C). Consistently, the expression of phosphorylated ERK was significantly increased after Reelin stimulated‐exosomes treatment (Figure 4F,G). To investigate how UBE2K regulates ERK, Co‐IP assays were performed in Lenti‐X 293T cells—a standard and widely accepted heterologous system for evaluatingprotein‐protein interactions. The results revealed interaction between UBE2K and ERK, specifically involving the ERK2 isoform (Figure 4H,I). To further provide orthogonal evidence at endogenous levels, high‐resolution confocal immunofluorescence showed that endogenous ERK co‐localized with UBE2K in MSCs (Figure 4J), establishing the spatial basis for their interaction under physiological conditions. Next, we tested whether this physical binding leads to UBE2K‐mediated ERK ubiquitination and activation. The results revealed that UBE2K overexpression increased Lys63‐linked ubiquitination of ERK, but not its Lys48‐linked ubiquitination (Figure 4K).

To evaluate whether Schwann cell‐derived UBE2K is essential for osteogenic differentiation, Schwann cells were transfected with a pool of three targeted siRNAs. UBE2K protein levels in both donor Schwann cells and their secreted exosomes were significantly reduced, as detected by Western blot (Figure S5D,E). Moreover, *Ube2k* knockdown in Schwann cells effectively inhibited pro‐osteogenic property of Reelin‐stimulated exosomes on MSCs, shown by decreased ALP and Alizarin Red staining (Figure S5F–H). Consistently, the osteogenic differentiation was also increased by UBE2K overexpression in MSCs (Figure S5I,J). Moreover, Reelin stimulated exosomes treatment increased ERK phosphorylation, whereas the ERK inhibitor U0126 suppressed both baseline p‐ERK levels and those induced by Reelin‐stimulated exosomes (Figure S5K). Moreover, ERK inhibitor U0126 treatment could partially abolish the promoting effects of Reelin stimulated‐exosomes for osteogenic differentiation of MSCs (Figure 4L,M). These findings indicate that Reelin‐stimulated exosomes promote osteogenic differentiation by activating ERK pathway, which is associated with Lys63‐linked polyubiquitination.

### 2.5 Reelin Deficiency Impairs Bone Defect Healing In Vivo

Given that *Reln^−/−^* mice exhibit severe neurodevelopmental malformations and potential early lethality [32], we employed*Reln^+/−^* mice and their wild‐type littermates to verify the role of Reelin in bone metabolism and regeneration. The results revealed that thinner calvarial bone was detected in *Reln^+/−^* mice (Figure 5A,B). Consistently, impaired skeletal structure with reduced bone volume/tissue volume (BV/TV) and BMD was observed compared to littermate controls (Figure 5C,D). To validate the pivotal role of Reelin in bone regeneration, calvarial defects were created in *Reln^+/−^* mice. A time‐course micro‐CT analysis at 0, 3, 7, and 28 days post‐injury showed no apparent difference in defect morphology between control and *Reln* ^+/−^ mice at 3 or 7 days, whereas bone regeneration was significantly impaired in *Reln* ^+/−^ mice at 28 days, evidenced by a marked reduction in bone volume and a larger unhealing area (Figure S6A–C, Figure 5E–G). Consistently, the histological analysis revealed superior bone formation in control mice compared with *Reln^+/−^* mice 28 days after calvarial bone defects (Figure 5H). Moreover, the expressions of osteogenic marker RUNX2 and myelin marker MBP were lower at the injury site in *Reln^+/−^* mice compared with control ones (Figure 5I–L). Together, these results verified that Reelin is involved in neurogenic bone regeneration.

**FIGURE 5:**
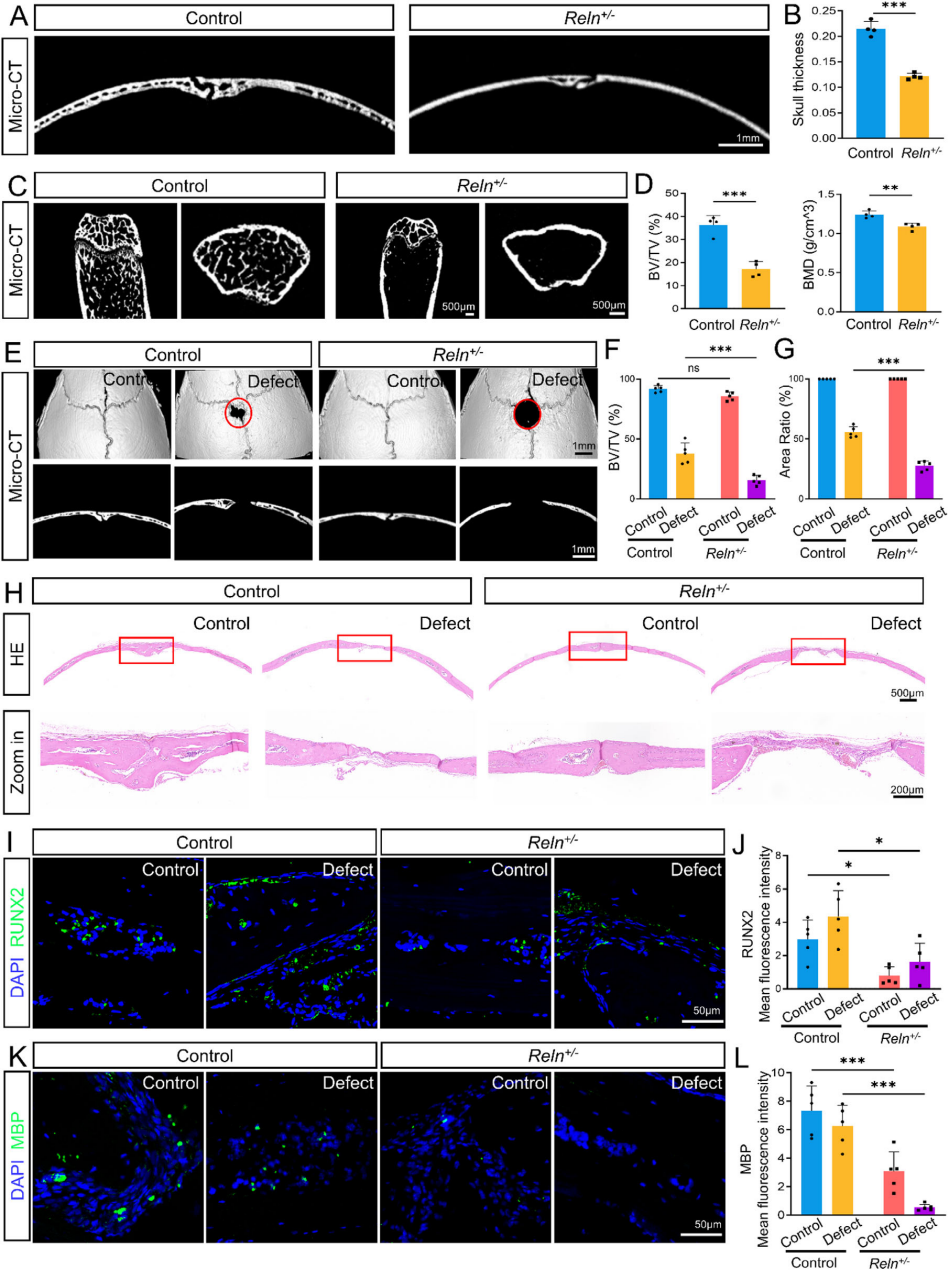
Reelin deficiency impairs bone defect healing in vivo. (A) Representative micro‐CT images (coronal view) of the calvarial bone in control and *Reln^+/−^* mice. (B) Quantitative analysis of calvarial thickness based on micro‐CT measurements. (C) Representative micro‐CT images of femur in control and *Reln^+/−^* mice. (D) Quantitative analysis of BV/TV and BMD based on micro‐CT measurements. (E) Representative 3D micro‐CT reconstructions and coronal 2D sections of the calvarial defects in control and *Reln^+/−^* mice at 4 weeks post‐injury. (F, G) Quantitative analysis of BV/TV and defect area ratio in regeneration area determined by micro‐CT measurements at 4 weeks post‐injury. (H) Representative images of H&E staining showing the histological features of the calvarial defects at 4 weeks post‐injury. (I, K) Representative immunofluorescence images of (I) RUNX2 and (K) MBP expression in calvarial bone defects of control and *Reln^+/−^* mice at 4 weeks post‐injury. (J, L) Quantitative analysis of the (J) RUNX2 and (L) MBP fluorescence intensity shown in (I, K). (*p < 0.05, **p < 0.01, ***p < 0.001). Each dot represents an individual replicate (*n* = 4 in B, D; *n* = 5 in F, G, J, L). Statistical significance was analyzed by unpaired Student's *t* test (B, D). Statistical significance was analyzed by Two‐way ANOVA with Bonferroni post‐hoc test (F, G, J, L).

### 2.6 Construction and Characterization of MSN‐PEI‐Based CRISPRa_*Reln* Delivery System In Vitro

To exploit the role of Reelin in bone regeneration for efficient and cost‐effective translational applications, we designed surface functionalized MSNs (MSN‐PEI) with radial porous structure to enable efficient loading of *Cas9* mRNA and gRNAs (MSN‐CRISPRa_*Reln*) (Figure 6A). Scanning and transmission electron microscopy (SEM and TEM) revealed that MSN‐PEI exhibited a uniform spherical morphology with a highly porous structure (Figure 6B). Measurement of 100 particles in TEM images gave a diameter of 125.5 ± 5.8 nm. The pore size distribution, calculated from the adsorption branch using the Barrett–Joyner‐Halenda (BJH) model, was centered at ∼7 nm (Figure 6C), consistent with efficient accommodation of nucleic acid cargos. Surface modification with polyethylenimine (PEI) resulted in a substantial shift in zeta potential from −36.5 to +45.6 mV, confirming successful charge reversal and enhanced electrostatic interaction with negatively charged RNA molecules (Figure 6D). Nitrogen adsorption‐desorption analysis displayed a characteristic type IV isotherm, indicative of mesoporosity, with a pronounced capillary condensation step at a relative pressure (P/P_0_) of ∼0.8 (Figure 6E). This functionalization was further validated by Fourier transform infrared (FTIR) spectroscopy, which showed characteristic absorption bands corresponding to the PEI backbone (Figure 6F). Dynamic light scattering (DLS) measurements showed hydrodynamic diameters of 152.3 ± 11.7 nm for MSN and 188.7 ± 10.1 nm for MSN‐PEI in water (mean ± SD; from 3 batches; Figure 6G). For MSN‐PEI, the hydrodynamic diameter was 197.2 ± 11.6 nm in DMEM and 216.0 ± 15.9 nm in DMEM containing 10% FBS (*n* = 3 batches per condition; Figure 6H). The larger diameter in serum‐containing medium may reflect protein adsorption.

**FIGURE 6:**
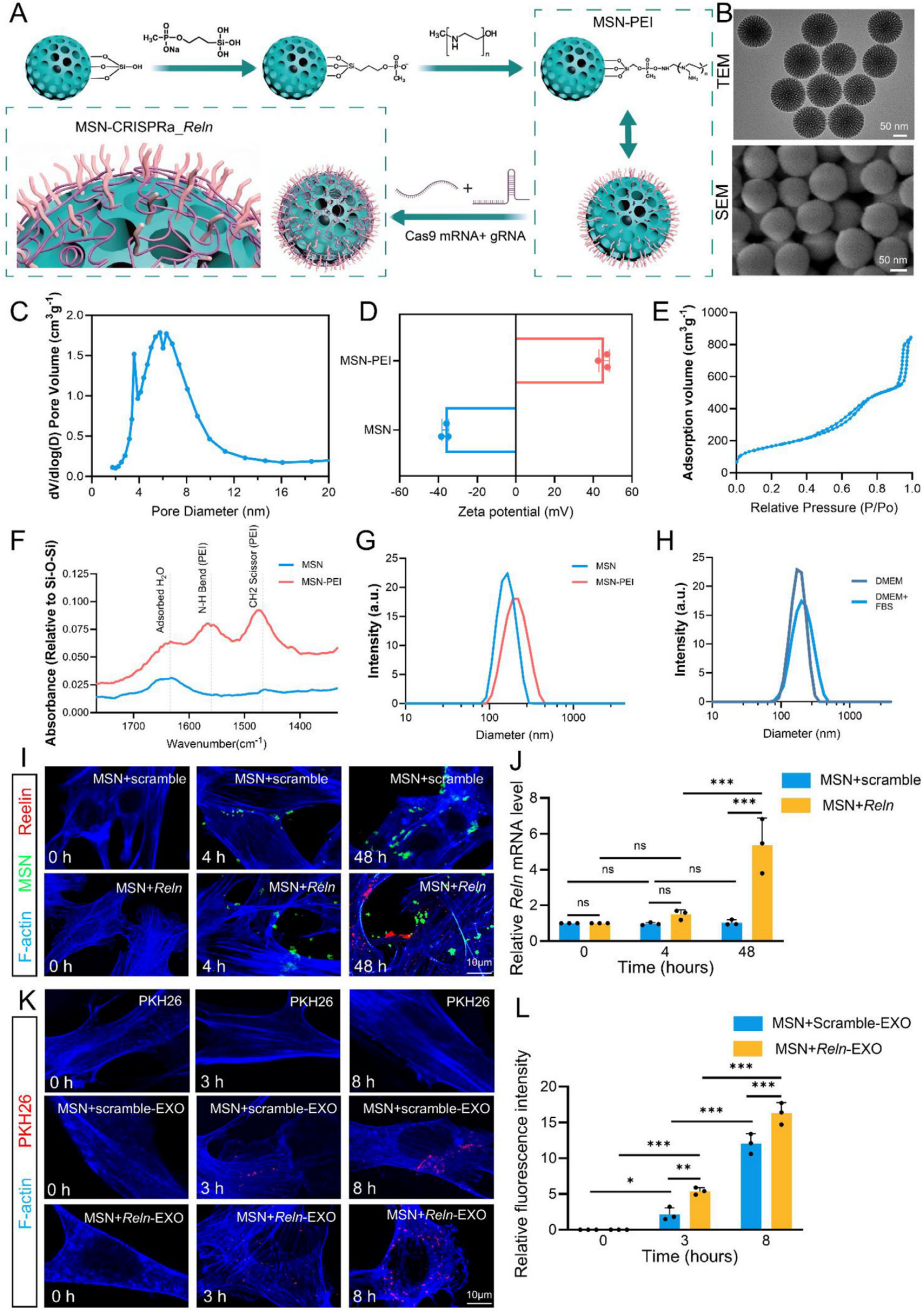
Construction and characterization of MSN‐PEI‐based CRISPRa_*Reln* delivery system in vitro. (A) Schematic diagram illustrating the loading of *Reln* gRNA and *Cas9* mRNA into MSNs. (B) Transmission electron microscopy (TEM) and scanning electron microscopy (SEM) images of MSN. (C) Barrett–Joyner–Halenda (BJH) pore size distribution of MSN. (D) Zeta potential of MSN and MSN‐PEI. (E) Nitrogen adsorption/desorption isotherm of MSN. (F) Fourier Transform Infrared (FTIR) analysis of MSN and MSN‐PEI. (G) Dynamic light‐scattering analysis of MSN and MSN‐PEI. (H) Dynamic light‐scattering analysis of MSN‐PEI in DMEM, and DMEM supplemented with FBS groups. (I) The MSN enrichment and expression of functional Reelin protein in Schwann cells after treatment by MSN‐CRISPRa_*Reln* for 0, 4, and 48 h. (J) The mRNA level of *Reln* in Schwann cells after treatment by MSN‐CRISPRa_*Reln* for 0, 4, and 48 h, analyzed by RT‐qPCR. (K, L) The exosomes endocytosis by MSCs after treated with exosomes derived from MSN and MSN‐CRISPRa_*Reln* treated Schwann cells, as assessed by immunofluorescence staining. (*p < 0.05, **p < 0.01, ***p < 0.001). Each dot represents one individual replicate (*n* = 3 in J, L). Statistical significance was analyzed by Two‐way ANOVA with Bonferroni post‐hoc test (J, L).

To optimize CRISPR activation efficiency, N1‐methylpseudouridine–modified *Cas9* mRNA and 2′‐O‐methyl–modified scramble gRNA or *Reln* gRNA were mixed at a molar ratio of 3:1 prior to loading into MSN‐PEI. Quantitative analysis revealed high encapsulation efficiency of MSN‐PEI for the *Cas9* mRNA and gRNA complex, reaching 69.60% ± 2.66% for CRISPRa_*Reln* and 66.89% ± 0.39% for the scramble group. TEM imaging with negative staining indicated that MSN‐PEI, MSN‐CRISPRa_scramble, and MSN‐CRISPRa_*Reln* all retained a well‐defined mesoporous structure and spherical morphology. The presence of darker contrast surrounding the silica framework in MSN‐CRISPRa_scramble and MSN‐CRISPRa_*Reln* indicates successful loading of nucleic acids into the nanoparticles (Figure S7A).

Furthermore, RNA protection evaluation demonstrated that free mRNA was completely degraded by 12 h (0% retention), whereas MSN‐PEI encapsulation maintained substantial RNA retention for both MSN‐CRISPRa_scramble (34.99% ± 4.69%) and MSN‐CRISPRa_*Reln* (31.17% ± 5.66%) at 12 h (Figure S7B), indicating the effective protection afforded by MSN‐PEI against enzymatic degradation. In the presence of 10% FBS, RNA complexes exhibited a faster release rate than in PBS alone, reaching a cumulative release of ∼60% at 72 h for both MSN‐CRISPRa_scramble and MSN‐CRISPRa_*Reln* (Figure S7C,D). Finally, we monitored the hydrodynamic diameter of MSN‐CRISPRa‐*Reln* nanoparticles during storage in DMEM supplemented with 10% FBS at 4°C. Measurements on days 1, 3, and 7 showed that the hydrodynamic diameter remained approximately 210 nm, with no marked change over the seven‐day period (Figure S7E). This result suggests no detectable increase in particle size under the storage conditions tested.

To evaluate in vitro cytocompatibility, CCK‐8 assays were performed at 24, 48, and 72 h, showing that neither unloaded MSN, MSN‐PEI, nor MSN‐CRISPRa transfection with or without *Reln* altered Schwann cell viability (Figure S7F–H). To visualize cellular uptake and subsequent protein expression, MSN‐CRISPRa_RNA was added to the culture medium of Schwann cells. MSN‐CRISPRa_RNA adhered to the surface of Schwann cells and was subsequently internalized (Video S1). Moreover, the internalization of MSN nanoparticles by Schwann cells was observed at 4 h post‐treatment, while *Reln* mRNA expression showed a 5.3‐fold increase in the MSN‐CRISPRa_*Reln* group at 48 h with no significant change in the scramble group, indicating *Reln* gRNA successful intracellular delivery and CRISPR activation (Figure 6I,J). Off‐target analysis allowing up to two mismatches predicted a single potential off‐target locus, located within an intronic region shared by *Tmem108* and *Gm32743* (chr9:103,402,595–103,402,616, GRCm39). The off‐target protospacer is 5′‐TCCGGGCACAGCTAGAGGTGGG‐3′. Further RT‐qPCR analysis revealed that MSN‐CRISPRa_*Reln* failed to alter the mRNA expression levels of *Tmem108* or *Gm32743* (Figure S8A,B). Because the dCas9‐VP64 fusion drives transcriptional activation specifically at promoter or enhancer regions [33, 34], binding at this intronic locus is insufficient to trigger off‐target gene expression, indicating the high specificity of our MSN‐CRISPRa_*Reln* system. Furthermore, the internalization of exosomes by MSCs was significantly enhanced in the MSN‐CRISPRa_*Reln*‐EXO group, as evidenced by the time‐dependent accumulation of PKH26 signals and a higher relative fluorescence intensity at both 3 and 8 h compared to the scramble‐EXO group (Figure 6K,L). Collectively, these results demonstrate that the MSN‐PEI platform serves as an efficient delivery vehicle for CRISPRa_*Reln*, enabling the stable intracellular transport and functional activation of Reelin.

### 2.7 MSNs‐Based *Reln* RNA Delivery Promotes Bone Regeneration Efficiently

To assess the therapeutic potential of MSN‐CRISPRa_*Reln* in vivo, we developed a localized delivery system by impregnating nanoparticles into gelatin sponges, which served as a functional scaffold (Figure S9A). Notably, MSN‐CRISPRa_*Reln* was effectively delivered to the bone defect area with functional Reelin protein expression, whereas no significant difference was observed among the control, MSN alone, and MSN‐CRISPRa_scramble groups (Figure 7A,B). The MSN‐CRISPRa_*Reln* promoted bone formation and accelerated bone repair in calvarial bone defect model compared to the control, MSN, and MSN‐CRISPRa_scramble groups, as assessed by micro‐CT and histological staining (Figure 7C–F). Due to the high incidence of fractures in clinical practice, next we analyzed the effects of MSN‐CRISPRa_*Reln* on bone fracture healing. In the untreated fracture and MSN‐CRISPRa_scramble groups, a complete lack of cortical bone continuity and a significant deterioration of the bone microarchitecture were observed (Figure S9B–F). In contrast, the continuity of cortical bone, bone volume fraction, and trabecular number were effectively restored after treatment with MSN‐CRISPRa_*Reln* compared with the control and scramble groups (Figure S9B–F). Moreover, the expression of RUNX2 and MBP was also significantly increased in the MSN‐CRISPRa_*Reln* treated group around the injured area (Figure 7G–J, Figure S9G,H). Together, these data underscore the efficacy of MSNs as a robust RNA delivery tool, enabling CRISPRa‐mediated *Reln* activation to promote bone regeneration by establishing a neurogenic microenvironment (Figure S10).

**FIGURE 7:**
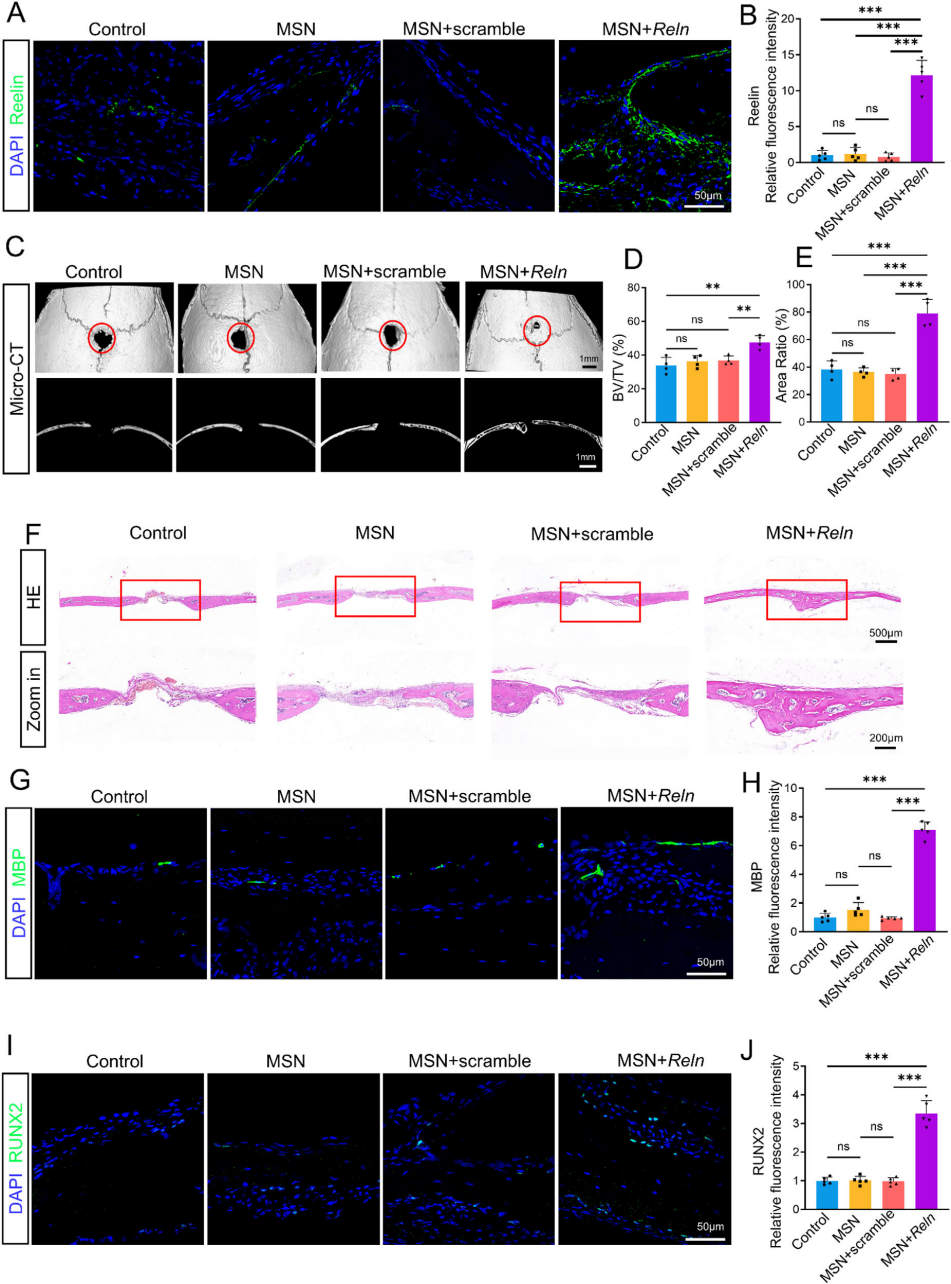
MSNs‐based RNA delivery promotes bone regeneration efficiently. (A, B) The expression of Reelin in the calvarial defect area at 4 weeks post‐injury following treatment with control, MSN, MSN + scramble, and MSN + *Reln*, as assessed by immunofluorescence staining. (C) Representative 3D micro‐CT reconstructions and 2D coronal sections of the calvarial defects in control, MSN, MSN + scramble, and MSN + *Reln* treated mice at 4 weeks post‐injury. (D, E) Quantitative analysis of bone volume fraction (BV/TV) and residual defect area ratio in control, MSN, MSN‐+scramble, and MSN + *Reln* treated mice. (F) H&E staining showing the histological features of the calvarial defects in control, MSN, MSN + scramble, and MSN + *Reln* treated groups at 4 weeks post‐injury. (G–J) The expression of RUNX2 (G) and MBP (I) at the defect site in control, MSN, MSN + scramble, and MSN + *Reln* treated groups at 4 weeks post‐injury. (**p < 0.01, ***p < 0.001). Each dot represents an independent replicate (*n* = 4 in (D), (E); *n* = 5in B, H, J). Statistical significance was analyzed by One‐way ANOVA with Bonferroni post‐hoc test (B, D, E, H, J).

## 3 Discussion

The regeneration of bone defects has long been recognized as a significant clinical challenge. In this study, we identified that Reelin was essential for bone regeneration. Functionally, Reelin could promote the communication between Schwann cells and MSCs by enhancing the exosomes from Schwann cells trafficking and internalization by MSCs. These exosomes deliver UBE2K, which promoted ERK pathway activation associated with K63‐linked polyubiquitination, thereby driving osteogenic differentiation. The crosstalk between Schwann cells and mesenchymal stem cells constitutes a central paradigm for coordinating nerve and bone repair. Reelin is reported to be secreted by neural and non‐neural cell types, including Cajal‐Retzius cells, GABAergic interneurons, granule cells, and brain endothelial cells [35], as well as hepatic stellate cells, myofibroblasts, and human hematopoietic stem cells [36]. Reelin is also found to be secreted by lymphatic endothelial cells [28]. The precise cellular origin of Reelin in the bone microenvironment requires further investigation. In the present study, a rapid migration of *Plp^+^* Schwann cells toward the defect margins was detected by day 3 post‐injury, reaching a peak by day 7. This process was accompanied by elevated expression of GFAP, MBP, and PGP9.5. These results indicate that Schwann cells serve as core components in the bone microenvironment reconstruction. Notably, the skeletal system is characterized by extensive innervation, which plays a pivotal role in bone injury repair and regeneration. A complex “nerve‐bone” regulatory axis is constructed by neuropeptides, neurotrophins, and the sympathetic and parasympathetic nervous systems, which precisely modulate bone regeneration either through direct interaction with target cell receptors or by indirectly tuning local inflammatory cytokine levels [37, 38, 39, 40]. While the recruitment of Schwann cells and osteogenic differentiation were highly synchronized on the temporal axis, recent evidence showed that Schwann cell‐derived extracellular vesicles attenuated bone resorption by activating PTEN to inhibit PI3K/AKT‐mediated osteoclastogenesis [41]. In multiple myeloma bone disease, Reelin depletion promoted osteoblast activity and inhibited osteolysis by suppressing tumor‐derived, pro‐osteolytic Reelin within the pathological tumor–bone microenvironment [42]. In this study, we revealed that Reelin within the regenerative niche drives MSC osteogenesis, suggesting that the biological effects of Reelin may be dependent on the cellular source and microenvironmental context.

Schwann cell‐derived exosomes are recognized for their pivotal roles in orchestrating nerve injury repair and regeneration. Peripheral nerve regeneration is promoted by mechanically stimulated Schwann cell‐derived extracellular vesicles through the transfer of miR‐23b‐3p [43]. Furthermore, Schwann cell‐derived exosomes combined with a methylprednisolone composite patch facilitate spinal cord injury repair by regulating the inflammatory microenvironment and enhancing neural regeneration [44]. Here, we demonstrate that Schwann cell‐derived exosomes act as key mediators in promoting MSC osteogenic differentiation, which is in accordance with previous reports [45, 46]. Moreover, nerve growth factor participates in early bone repair by activating p75 signaling in mesenchymal osteogenic precursors and coordinating their migration toward the injury site [47]. Similarly, brain‐derived neurotrophic factor promotes osteoblast differentiation through TrkB‐dependent signaling, leading to increased ALP activity, matrix mineralization, and osteogenic marker expression [48]. These findings underscore the role of Schwann cells as a functional neural bridge that delivers neurogenic signals regulating MSCs, which facilitate bone defect healing.

Exosomal function is modulated through the manipulation of its secretion or uptake [49, 50]. We found that Reelin did not significantly alter the total secretion or physical properties of Schwann cell‐derived exosomes, but potently enhanced their endocytosis by MSCs. Mechanistically, proteomic profiling revealed a significant enrichment of UBE2K in Reelin‐stimulated exosomes. Moreover, UBE2K mediates the enhancement of K63‐linked polyubiquitination of ERK, but not K48‐linked modification. The K63‐linked ubiquitination leads to ERK activation, which drives the osteogenic differentiation of MSCs. As an E2 conjugating enzyme, it likely acts in tandem with an E3 ubiquitin ligase to fine‐tune this process. Identifying the specific E3 ligase, which participates in this process needs further investigation.

For translational application, we developed an MSN‐based CRISPRa delivery system capable of driving local *Reln* expression at the defect area to trigger Reelin expression, which promotes neurogenic bone regeneration. It is reported that upregulation of endogenous Sox trio (*Sox5/6/9*) via gRNA arrays can drive MSC differentiation and enhance bone healing. However, that approach relied on viral vector‐mediated delivery of dCas9‐encoding DNA, which has risks of genomic integration and prolonged *Cas9* activity [51]. Delivering *Cas9* in mRNA form enables transient and controllable gene activation without integration concerns, which has been used in oncology and antiviral therapy [17, 18]. To our knowledge, this study is the first to report the application of *Cas9* mRNA‐mediated CRISPR activation of *Reln* in bone repair. Notably, although elevated Reelin protein expression remained detectable at the defect site at 4 weeks after local administration of *Reln* mRNA, we did not determine how long the delivered Cas9 mRNA/gRNA activity persisted in vivo. The Reelin signal may reflect sustained endogenous Reelin expression or secondary cellular responses initiated by *Reln* activation rather than prolonged persistence of the RNA cargos themselves. Future in vivo time‐course studies measuring *Cas9* mRNA/gRNA, *Reln* transcription, and Reelin protein at early and intermediate time points need to be further studied.

Various mRNA delivery strategies have been developed. Lipid nanoparticles provide high transfection efficiency, but exhibit structural instability under local inflammatory or acidic tissue microenvironments [21]. Biomimetic exosomes offer superior biocompatibility, but their application is hindered by low loading efficiency for large mRNA transcripts like *Cas9* mRNA [52]. Inorganic MSNs feature rigid architectures that physically protect RNA from enzymatic degradation [53], while PEI surface functionalization enables efficient co‐loading and delivery of both *Cas9* mRNA and *Reln* gRNA. These structural and functional advantages suggest that MSNs effectively overcome the microenvironmental and cargo‐size limitations of conventional delivery platforms. Furthermore, we showed that these MSNs are effectively internalized by Schwann cells, which enables the targeted intracellular release of the CRISPR activation components. This MSN‐based system effectively delivered CRISPR components both in vitro and in vivo, which enhanced the repair of calvarial and tibial defects. The degradation process of locally delivered MSNs may proceed via macrophage cellular uptake and gradual dissolution, releasing silicon ions [54]. Meanwhile, intracellular MSN degradation products promote autophagy to enhance particle clearance and establish a pro‐osteogenic osteoimmune microenvironment [55]. However, further study needs to be done to explore the exact process for the MSN degradation in the bone.

There are several limitations of this study. First, Schwann cell‐derived EVs were isolated using a polymer‐based precipitation method. Although the isolated fractions exhibited typical vesicular morphology and size distribution, and expressed established EV‐positive markers, the polymer‐based precipitation process may also isolate soluble proteins, lipoproteins, and other non‐vesicular particles. Future studies using higher‐specificity isolation approaches, such as size‐exclusion chromatography or density‐gradient ultracentrifugation, may further improve EV purity and molecular characterization. Second, although elevated Reelin protein expression remained detectable at the defect site at 4 weeks after a single local administration of MSN‐CRISPRa_*Reln*, the present study did not determine how long the delivered *Cas9* mRNA/gRNA or CRISPRa activity persisted in vivo. The 4‐week Reelin signal may therefore reflect sustained endogenous Reelin expression or secondary cellular responses initiated by early *Reln* activation rather than prolonged persistence of the RNA cargos themselves. Future in vivo time‐course studies measuring *Cas9* mRNA/gRNA, *Reln* transcription, and Reelin protein at early and intermediate time points will be required to clarify this temporal relationship. Third, UBE2K knockdown was performed using a pooled siRNA strategy, and the knockdown efficiency was validated at the protein level in donor Schwann cells and their secreted exosomes. A gain‐of‐function experiment with pCMV‐UBE2K overexpression was also performed, which promoted the osteogenic differentiation of MSCs. However, this construct was not siRNA‐resistant, and a formal add‐back rescue with an siRNA‐resistant *Ube2k* plasmid will be performed in future studies. Fourth, only male mice were used in our in vivo experiments. Potential sex‐dependent effects in Reelin‐mediated bone regeneration need to be further evaluated in female models. Fifth, although particle size was monitored over seven days, RNA integrity following storage was not directly assessed. Further studies are required to determine whether the formulation preserves intact RNA and its biological activity during storage.

Collectively, our study demonstrates that Reelin promotes bone regeneration by controlling the crosstalk between Schwann cells and MSCs, which establishes a neurogenic bone regeneration microenvironment. Functionally, we developed MSN‐CRISPRa_*Reln* as a potent RNA delivery platform for bone regeneration, pending further biosafety validation.

## 4 Materials and Methods

### 4.1 Animals

Male C57BL/6J mice (8–10 weeks, 20–25 g) were purchased from Vital River Laboratory Animal Technology (Beijing, China). *Reln* ^+/−^ (T009126, C57BL/6J background, 8–10 weeks) male mice were obtained from GemPharmatech (Nanjing, China). *Plp‐Cre^ERT2^* (JAX: 005975), *DTA* (JAX: 009669), and *Ai14* (JAX: 007914) mice were purchased from the Jackson Laboratory (USA). All mice were maintained under SPF conditions at Peking University School and Hospital of Stomatology. Animal experiments were approved by the Institutional Animal Care and Use Committee of Peking University (No. LA2022476). Experiments were performed in accordance with ARRIVE guidelines.

*Plp‐Cre^ERT2^* mice were crossed with *Ai14* mice to generate *Plp‐Cre^ERT2^*; *Ai14* mice, which were further bred with *DTA* mice to obtain *Plp‐Cre^ERT2^*; *Ai14*; *DTA* mice. Genotypes were confirmed by tail snip PCR. Tamoxifen (20 mg/mL in corn oil) was administered intraperitoneally (75 mg/kg/day for 3 days) to induce recombination. The ablation efficiency for *Plp‐Cre^ERT2^; Ai14; DTA* mice was quantified by measuring the proportion of red fluorescent cells under uninjured baseline conditions relative to *Plp‐Cre^ERT2^; Ai14* control mice (*n* = 4 independent mice per group).

For genotype‐based experiments, animals were assigned to groups according to genotype, whereas animals subjected to different injury or therapeutic interventions were randomly allocated to the corresponding experimental groups. Quantitative analyses, including micro‐CT measurements, histological evaluation, and immunofluorescence quantification, were performed by investigators blinded to genotype or treatment allocation.

No statistical methods were used to predetermine sample sizes. Sample sizes were selected based on previous studies using comparable murine bone‐injury models, preliminary experiments, and our previous experience with similar experiments. Exact sample sizes are provided in the corresponding figure legends, and n represents the number of independent animals for all in vivo experiments.

Predefined exclusion criteria included unexpected perioperative death, failure to establish the intended injury model, or technical failure that prevented reliable quantitative analysis. No animals or data points were excluded solely on the basis of the magnitude of the measured values.

### 4.2 The Reagents and Antibodies

The anti‐RUNX2 (12556), anti‐Erk1/2 (4695), and anti‐p‐Erk1/2 (4370) antibodies were purchased from Cell Signal Technology (Beverly, MA, USA). The anti‐Erk1 (A19561), β‐actin (AC004), and anti‐Ubiquitin K48‐linkage (A3606) were purchased from Abclonal (Wuhan, China). The anti‐Erk1/2 (66192‐1‐Ig), anti‐UBE2K (11834‐3‐AP) were purchased from Proteintech (Wuhan, China). The anti‐Ubiquitin K63‐linkage (T56579S) was purchased from Abmart (Shanghai, China). The anti‐CD81 (sc‐166029) and anti‐TSG101 (sc‐7964) were purchased from Santa Cruz Biotechnology (Santa Cruz, CA, USA). The anti‐Reelin (ab78540), anti‐MBP (ab7349), anti‐PGP9.5 (ab108986), anti‐GFAP (ab68428), and anti‐CD146 (ab75769) were purchased from Abcam (Waltham, MA, USA). The Calcein Violet 450 AM (65‐0854‐39) was purchased from Invitrogen (Carlsbad, CA, USA). pCMV‐ERK1‐FLAG (RC204196) was purchased from Origene (Wuxi, China). pCMV‐ERK2‐FLAG (HG10030‐CF), pCMV‐UBE2K‐Myc (HG16296‐CM) were purchased from Sino Biological (Beijing, China).

### 4.3 Cell Culture

Bone marrow MSCs were isolated from femurs of C57BL/6J mice as described previously [56]. Briefly, bone marrow was flushed from femurs of 8 weeks old mice using PBS containing 10% penicillin/streptomycin. After centrifugation, cells were resuspended in α‐MEM supplemented with 20% fetal bovine serum (FBS, Wuhan Procell Biotechnology Co., Ltd.) and 1% penicillin/streptomycin. The single‐cell suspension was plated in 10‐cm culture dishes and maintained at 37°C under 5% CO_2_. Non‐adherent cells were removed 48 h later, and the medium was refreshed every 3–5 days. Second passage mouse MSCs were used for subsequent experiments.

The Schwann cells (BFN60807359) were purchased from the Shanghai Cell Bank of Chinese Academy of Sciences (China). Cells were cultured in high‐glucose DMEM supplemented with 10% FBS and 1% penicillin–streptomycin at 37°C with 5% CO_2_. Medium was refreshed every 1‐2 days. Cells were passaged at 1:3 when 70%–80% confluent using 0.25% trypsin–EDTA.

### 4.4 HE Staining

For HE staining, sections were immersed in hematoxylin solution for 5 min, followed by a rinse with deionized water. Subsequently, the sections were re‐stained with eosin for 3 min and then immersed in deionized water for 1 min. Finally, the specimen was mounted with neutral resin and cover glasses.

### 4.5 Tissue and Cell Immunofluorescence

The samples were collected for sectioning and subsequently preserved using a 4% paraformaldehyde solution. The 1 × casein blocking solution was used at room temperature for 60 min. Following this, the primary antibody (1:100) was applied and incubated at 4°C. Secondary antibody incubation was performed at room temperature for 1 h. Imaging was performed by laser‐scanning confocal microscopy on a Leica SP8 inverted microscope.

### 4.6 TESOS Tissue Clearing and Deep Imaging

TESOS tissue clearing was performed as previously described [57, 58]. Tissue samples were fixed in 4% PFA at 4°C for 24 h. The skeletal tissues were sequentially incubated in 0.5 M EDTA for 7 days. Subsequently, the samples were sequential incubation in Quadrol solution for two days at room temperature. This was followed by a gradient treatment for three days using 30%, 50%, and 70% TB delipidation solutions. Finally, the tissues were made transparent through immersion in BB‐BED medium at 37°C for 2 days. Upon achieving transparency, samples were embedded via high‐power UV irradiation on ice. 3D imaging was performed with a Leica SP8 confocal microscope.

### 4.7 Micro‐CT Analysis

Micro‐CT scans were performed with a high‐resolution Micro‐CT (PINGSENG, China) under the conditions of 90 kV and an isotropic voxel size of 15 µm. 3D reconstruction and subsequent analysis were conducted using the manufacturer's Avatar software (PINGSENG). For trabecular bone assessment, regions of interest were manually delineated at intervals of five slices. The primary morphometric parameter calculated was the bone volume to tissue volume ratio (BV/TV). The bone regeneration capacity was assessed by measuring the area of newly formed bone.

### 4.8 Osteogenic Differentiation and Staining

The osteogenic differentiation was induced as previously report [59]. Following induction for 14 days, MSCs were fixed with 4% paraformaldehyde for mineralization assessment. The fixed cells were then stained with 1% Alizarin Red solution (Beyotime, China) on a shaker for 5–10 min at room temperature and washed three times with DEPC water. For ALP activity, cells were induced for 7 days, fixed, and incubated with the BCIP/NBT substrate (Beyotime, China) as per the kit instructions for 1 h at room temperature. All stained samples were observed and imaged under an optical microscope.

### 4.9 Exosome Isolation and Characterization

Total exosome isolation was performed using the Total Exosome Isolation Kit (OriCell, China). Briefly, conditioned media were collected and sequentially centrifuged at 300 × *g* for 10 min, 3000 × *g* for 10 min, and 10 000 × *g* for 30 min to sequentially remove intact cells, cellular debris, and larger microvesicles. The filtered supernatant was thoroughly mixed with exosomes isolation reagent at a 4:1 (v/v) ratio and incubated overnight at 4°C. The mixture was subsequently centrifuged at 10 000 × *g* for 60 min at 4°C to yield exosome pellets. The purified exosomes were then analyzed by nanoparticle tracking analysis (ZetaView PMX 110). The particle concentrations of Exo and R‐Exo were 5.73 × 10^8^ ± 2.07 × 10^7^and 5.80 × 10^8^ ± 3.23 × 10^7^particles/mL, respectively, while the corresponding protein concentrations were 2.26 and 2.31 mg/mL. The calculated particle‐to‐protein ratios were therefore approximately 2.54 × 10^5^and 2.51 × 10^5^particles/µg protein for Exo and R‐Exo, respectively.

To prepare Reelin‐stimulated exosomes (R‐Exo), Schwann cells were incubated with 1 µg/mL recombinant Reelin protein (reconstituted in sterile PBS; YBio Biotechnology Co., Ltd., China; Cat. No. YBC775Mu01) for 24 h. For the control group (Exo), cells were treated with an equivalent volume of sterile PBS vehicle for 24 h under identical conditions. The conditioned media from both groups were then collected, and exosomes were isolated as described above. For all subsequent MSC treatments, exosomes were applied at a final concentration of 50 µg/mL (based on total protein quantified by BCA), unless otherwise indicated.

To inhibit ERK signaling, MSCs were pretreated with 10 µM U0126 (MedChemExpress, USA) or an equivalent volume of DMSO (vehicle control) for 1 h prior to R‐Exo administration. The inhibitor was maintained in the culture medium throughout subsequent treatments.

### 4.10 PKH26 Labeling

The exosomes were diluted in Diluent C to a final volume of 1 mL, followed by the addition of 6 µL PKH‐26 dye. The suspension was gently mixed and incubated for 5 min at room temperature. The labeling reaction was quenched by adding 2 mL of 10% BSA, after which culture medium was added to adjust the total volume to 8.5 mL. The labeled exosome suspension was then carefully layered onto 1.5 mL of 0.971 M sucrose solution and ultracentrifuged at 190 000 × *g* for 2 h at 4°C to remove excess dye. After centrifugation, the supernatant was discarded, and the exosome pellet was resuspended in PBS. For protein quantification, exosomes were lysed with RIPA buffer and measured by a BCA assay. Exosomes at 50 µg/mL were used for subsequent experiments. To exclude potential interference from non‐specific free dye aggregates, a PKH26 dye control was processed in parallel using the exact same protocol, with an equal volume of PBS used in place of exosomes. All fluorescence imaging was performed using identical exposure and acquisition settings across groups, and the background fluorescence intensity of the dye control was subtracted as baseline during quantitative analysis.

### 4.11 RT‐qPCR

Total RNA was extracted with Trizol reagent, and its concentration was determined using NanoDrop 2000 (Thermo Fisher, USA). According to the manufacturer's protocol, the RNA was reverse‐transcribed into complementary DNA (cDNA). Quantitative real‐time PCR (RT‐qPCR) was then carried out with 2× SYBR Green mix (Invitrogen, USA) on an Applied Biosystems Q3 Real‐Time PCR System to evaluate gene expression. All primer sequences were provided in Table S1.

### 4.12 Western Blot and Co‐Immunoprecipitation

Protein extracts for standard Western blot were prepared by lysing cells in ice‐cold RIPA buffer, followed by centrifugation at 12 000 rpm (4°C, 30 min) to collect the supernatant. For exogenous Co‐IP assays, Lenti‐X 293T cells cultured in 10‐cm dishes were co‐transfected with specified combinations of plasmid vectors (3 µg each for pCMV‐ERK1‐Flag, pCMV‐ERK2‐Flag, or pCMV‐UBE2K). After 48 h, cells were harvested and lysed in 1 mL cell lysis buffer (CST, #9803). The cleared supernatant was collected, from which a 20 µL aliquot was reserved as the input control. The remaining lysate was equally divided and incubated with 3 µg of normal control IgG or 3 µg of specific anti‐Flag/anti‐Myc primary antibodies under gentle rotation at 4°C or 12 h. Subsequently, 20 µL of protein A/G agarose beads (Santa Cruz Biotechnology) were added and rotated for an additional 2 h at 4°C. For endogenous ubiquitination IP, MSCs cultured in 10‐cm dishes were transfected with 10 µg vector or UBE2K‐overexpression plasmids for 48 h and pretreated with MG132 (10 µM) for 2 h prior to harvesting. Lysates were immunoprecipitated with 3 µg of control IgG or anti‐ERK antibody following the same procedure as exogenous Co‐IP. Immunoprecipitated complexes were washed thoroughly, eluted, and denatured in SDS loading buffer at 100°C for 5 min. Denatured protein samples (inputs and IP eluates) were separated by SDS‐PAGE and transferred onto pre‐activated PVDF membranes. Membranes were blocked at room temperature for 1 h, incubated with primary antibodies (1:1000) overnight at 4°C, and then probed with HRP‐conjugated secondary antibodies (1:5000) for 1 h at room temperature. Protein signals were visualized using enhanced chemiluminescence (ECL) detection reagents.

### 4.13 Gene Silencing

Schwann cells were seeded in 10 cm dishes. The next day, after the cells were attached, the corresponding siRNA (50 nM final concentration) was transfected for 48 h using the Lipofectamine 2000 according to the instructions, with 10 µL of each siRNA (1#, 2#, and 3#) per dish (30 µL total). Mouse *Ube2k* siRNA and negative control siRNA were generated by Genscript. The siRNA pool containing three target sequences was listed as follows: *Ube2k* siRNA

1# GUGGAAGAUAUCAACUAGAdTdT,
2# CUAUGGGUUUUGAUAGGAAdTdT,
3# GGUCCGGUUUAUCACUAAAdTdT.

### 4.14 Phalloidin Staining

The cells were fixed and stained with FITC‐conjugated phalloidin for 30 min as previously described [60]. The staining of cytoskeleton and nucleus were observed by OLYMPUS IX73 inverted microscope (Tokyo, Japan).

### 4.15 Single‐Cell RNA Sequencing Data Processing and Cell–Cell Communication Network Analysis

Bone marrow‐derived scRNA‐seq data from two experimental groups (UNJ, INJ7) were obtained from GEO (GSE245094). Data processing was performed using the Seurat package (v5.3.1) in R. Raw count matrices for each sample were loaded, and initial Seurat objects were created, filtering out genes detected in fewer than 5 cells and cells with fewer than 300 detected genes. The two objects were merged into a single dataset. Potential doublets were identified and removed using the DoubletFinder algorithm (v2.0.6) separately for each sample. Quality control metrics were calculated for the merged object, including the percentage of mitochondrial gene expression (percent.mt) and hemoglobin gene expression (percent.HB). Cells were filtered based on the following criteria: number of detected genes (nFeature_RNA) between 500 and 10 000, and mitochondrial percentage (percent.mt) below 25%. Data normalization (NormalizeData), identification of highly variable features (FindVariableFeatures), and scaling (ScaleData) were performed.

To correct for batch effects between samples, Harmony (v1.2.3) integration was applied using orig.ident as the batch variable. Dimensionality reduction was performed on the integrated data using UMAP (30 Harmony components). Cells were clustered using a shared nearest neighbor (SNN) modularity optimization‐based algorithm (FindNeighbors and FindClusters, resolution = 0.5) on the Harmony embeddings. Cell clusters were manually annotated into distinct cell types based on the expression of canonical marker genes visualized in a dot plot. All visualizations (UMAP, dot plot) were generated using Seurat functions.

Cell–cell communication networks were constructed separately for the UNJ and INJ7 conditions using the CellChat package (v1.6.1) in R. For each condition, the normalized expression matrix (slot = “data”) and cell type annotations were extracted from the integrated Seurat object.

The mouse ligand‐receptor interaction database (CellChatDB.mouse) was applied. CellChat then identified over‐expressed genes and over‐expressed ligand‐receptor interactions within each dataset. The communication probabilities between cell types were computed using a trimean approach (computeCommunProb), incorporating the effect of cell population size. These probabilities were aggregated at the signaling pathway level (computeCommunProbPathway). An aggregated communication network was derived (aggregateNet), and network centrality measures (e.g., degree centrality) were calculated for each signaling pathway (netAnalysis_computeCentrality) to infer key senders, receivers, and mediators.

To compare communication networks between conditions, the two CellChat objects were merged using the mergeCellChat function. The total number and strength of interactions were compared visually. Differential network analysis was performed to identify interaction pairs that were significantly altered between UNJ and INJ7. The results were visualized as differential circular network plots (netVisual_diffInteraction), and a differential interaction heatmap (netVisual_heatmap).

### 4.16 Bulk RNA‐Sequencing Analysis

RNA sequencing was performed on MSCs incubated with or without exosomes for 24 h, with three biological replicates per group (Beijing Genomics Institution). Sequencing libraries were prepared from 1 µg total RNA per sample using the NEBNext Ultra RNA Library Prep Kit, followed by sequencing on an Illumina HiSeq 2500 platform with the TruSeq SBS‐Kit v2 (200 cycles). Differentially expressed genes (DEGs) were identified using thresholds of |log_2_Fold Change| ≥ 1 and adjusted P ≤ 0.05. Functional enrichment analysis of DEGs was subsequently carried out using the Kyoto Encyclopedia of Genes and Genomes (KEGG) and Gene Ontology (GO) databases to identify associated biological pathways.

### 4.17 Mass Spectrometry

Exosomes were lysed in 4% (w/v) sodium deoxycholate (SDC) buffer at 95°C for 5 min to ensure complete protein denaturation and protease inactivation. Samples were homogenized via sonication at 4°C. Proteins were reduced and alkylated with a reduction/alkylation buffer for 30 min at 25°C (1,500 r.p.m.). Trypsin digestion was performed overnight at 37°C (enzyme‐to‐substrate ratio of 1:50, w/w). The reaction was quenched by acidification with 1% trifluoroacetic acid (TFA), followed by centrifugation to remove debris. Peptides were desalted using SDB‐RPS StageTips and dried via vacuum centrifugation. Peptides were separated on a home‐packed C18 capillary column (1.9 µm, 120 Å; Dr. Maisch) using a Vanquish Neo UHPLC system coupled to an Orbitrap Ascend Tribrid mass spectrometer. Separation was performed over a 120‐min gradient (400 nL/min) using 0.1% formic acid in water (mobile phase A) and 0.1% formic acid in 80% acetonitrile (mobile phase B). Data acquisition was performed in data‐dependent mode (Xcalibur 4.1) with full MS scans (m/z 350–1600) at 120 000 resolution, followed by top‐speed MS/MS scans. MS/MS spectra were processed via Proteome Discoverer (v.2.5, Thermo Scientific) against the mouse protein database. Search criteria included: full tryptic specificity, maximum two missed cleavages, carbamidomethylation of cysteine (fixed), and methionine oxidation (variable). Mass tolerances were 20 ppm for precursor ions and 0.02 Da for‐fragment ions. A false discovery rate (FDR) < 1% was applied for high‐confidence identification, and only unique peptides were utilized for protein quantification.

### 4.18 Synthesis and Characterization of MSN‐PEI Nanoparticles

MSNs were synthesized using a surfactant‐templated approach. Briefly, 60 mL of deionized water was heated to 60°C in a 250 mL round‐bottom flask under gentle stirring, followed by the addition of cetyltrimethylammonium bromide (CTAB, 3.0 g) and triethanolamine (TEA, 0.15 mL). The mixture was stirred for 1 h to ensure complete dissolution. Subsequently, 20 mL of cyclohexane containing 10% (v/v) tetraethyl orthosilicate (TEOS) was added dropwise, and the reaction was maintained at 60°C for 24 h under continuous stirring. The resulting particles were collected by centrifugation, washed repeatedly with ethanol and deionized water, and subjected to two consecutive template extraction steps using HCl/ethanol at 60°C for 24 h each.

For surface functionalization, the synthesized MSNs were first dispersed in water, and the pH was adjusted to 10. N‐(3‐(trimethoxysilyl)propyl)ethylenediamine was then added, and the reaction was carried out at 40°C for 2 h. The product was collected by centrifugation, washed thoroughly, and subsequently resuspended in a polyethyleneimine (PEI) solution prepared in carbonate buffer (pH 9.6). After stirring at room temperature for 4 h, the final MSN‐PEI nanoparticles were washed extensively with deionized water and air‐dried.

Morphological characterization was performed using scanning electron microscopy (SEM; Zeiss Sigma VP HD, Zeiss) and transmission electron microscopy (FEI Tecnai T12) operated at 120 kV. For SEM analysis, samples were dispersed in ethanol, drop‐cast onto aluminum foil, dried under vacuum, and sputter‐coated with a ∼3 nm layer of gold prior to imaging.

Nanoparticle morphology and dry diameter were examined by transmission electron microscopy (TEM; FEI Tecnai T12) operated at 120 kV. For quantitative size analysis, 100 individual nanoparticles were measured from TEM images acquired across at least four different grid squares. Results are reported as mean ± standard deviation (SD; *n* = 100 particles).

Hydrodynamic diameter was measured by dynamic light scattering (DLS; Malvern Zetasizer Advance Series‐Pro) at 25°C. Nanoparticles were dispersed in the specified solution and sonicated in an ultrasonic bath before measurement. MSN and MSN‐PEI were measured in water; MSN‐PEI was additionally measured in DMEM and DMEM supplemented with 10% fetal bovine serum (FBS). Three independently prepared nanoparticle batches were measured for each condition. Results are reported as mean ± SD of the three batch‐level measurements (*n* = 3 batches).

For storage test, freshly prepared MSN‐CRISPRa‐*Reln* nanoparticles were dispersed in DMEM supplemented with 10% FBS and stored at 4°C for 7 days. On days 1, 3, and 7, samples were gently resuspended before measurement. Hydrodynamic diameter was measured at 25°C by dynamic light scattering using a Malvern Zetasizer Advance Series‐Pro.

Nitrogen adsorption–desorption isotherms were acquired at −196°C using a Micromeritics Tristar II system. Samples were degassed at 100°C overnight before measurement. The specific surface area was calculated using the Brunauer‐Emmett‐Teller (BET) method, total pore volume was estimated from the adsorption capacity at a relative pressure (P/P_0_) of 0.99, and pore size distribution was determined from the adsorption branch using the Barrett‐Joyner‐Halenda (BJH) model.

Hydrodynamic size was measured by dynamic light scattering (DLS) at 25°C using a Malvern Zetasizer Advance Series‐Pro instrument.

For the preparation of MSN‐CRISPRa_gRNA, *Cas9* mRNA and gRNA were complexed at a molar ratio of 3:1 (0.45 nmol/µL *Cas9* mRNA: 0.15 nmol/µL gRNA) by incubation at 37°C for 10 min. This complex was then combined with MSN (20 µg/µL) at a volume ratio of 2:1 and incubated for an additional 10 min at 37°C to facilitate electrostatic adsorption and pore loading. MSN‐CRISPRa_gRNA (80 µg/mL) was used for in vitro experiments. gRNA sequence: *Reln*: TCCGGGCACAGCCCGAGGTG, scramble: ACGGAGGCTAAGCGTCGCAA.

### 4.19 RNA Release Kinetics

To evaluate the in vitro release kinetics of RNA, MSN‐CRISPRa_scramble and MSN‐CRISPRa_*Reln* nanoparticles were suspended in PBS or PBS containing 10% FBS, and gently shaken at 37°C. The RNA concentration in the supernatant was detected using a Qubit 4 Fluorometer with Equalbit RNA BR Assay Kit at 0, 1, 2, 6, 12, 24, 48, and 72 h.

### 4.20 RNA Retention Assay

To evaluate the protective ability of nanoparticles, free RNA, MSN‐CRISPRa_scramble, and MSN‐CRISPRa_*Reln* were placed in DMEM medium containing 10% FBS and incubated at 37°C for 0, 6, 12, and 24 h. 1% (w/v) SDS was added immediately to terminate the nuclease reaction and promote the dissociation of the vectors. To analyze the changes in RNA retention rate over time, equal volumes of samples from each group (corresponding to the initial total RNA of 200 ng at 0 h) were taken and mixed with formamide‐based RNA loading buffer. The samples were heated at 65°C for 10 min for denaturation, and then immediately quenched on ice for 3 min to completely disrupt the secondary structure. The samples were then loaded onto 1% agarose gel for electrophoresis to assess the stability of intact mRNAs and gRNAs.

### 4.21 Encapsulation Efficiency Analysis

To determine the RNA encapsulation efficiency of MSN‐PEI nanoparticles, the concentration of initial input RNA (C_initial_) and the unencapsulated free RNA remaining in the supernatant after nanoparticle binding (C_free_) were measured using a Qubit 4 fluorometer and an Equalbit RNA BR Assay Kit (Vazyme, China). The encapsulation efficiency was calculated using the following formula:

$$\mathrm{Encapsulation}\;\mathrm{Efficiency}\;(\%)\;=\frac{C_{\mathrm{initial}}-C_{\mathrm{free}}}{C_{\mathrm{initial}}}\;\times 100\%$$

### 4.22 CCK‐8 Cytotoxicity Assay

Cell cytotoxicity was evaluated using a CCK‐8 kit (Dojindo, Japan) at 24, 48, and 72 h post‐treatment. Schwann cells were seeded in 96‐well plates and randomly assigned to five groups: Control (untreated), bare MSN, unloaded MSN‐PEI, MSN‐PEI_scramble, and MSN‐PEI_*Reln*. At each time point, the cells were washed twice with PBS to remove residual nanoparticles. Subsequently, the medium was replaced with a fresh CCK‐8 working solution (10 µL CCK‐8 solution per 100 µL of fresh culture medium per well). The cells were then incubated in a 5% CO_2_ incubator at 37°C for 1 h in the dark. Subsequently, the absorbance at a wavelength of 450 nm was measured using a microplate reader.

### 4.23 Storage Stability of MSN‐CRISPRa_*Reln*

MSN‐CRISPRa_*Reln* nanoparticles were freshly prepared and stored at serum contained culture medium at 4°C for 7 days. At predetermined time points (Days 1, 3, and 7), the nanoparticles were gently resuspended, and their hydrodynamic particle size and polydispersity index (PDI) were measured using dynamic light scattering (DLS).

### 4.24 Calvarial Defect Model and Tibia Fracture Model

For calvarial defect model, 8‐10 weeks old mice were anesthetized with tribromoethanol, and 1.5 mm defects were created in the calvarium. Samples were harvested 4 weeks post‐surgery for analysis.

For the tibial fracture model, anesthetized mice were prepared by shaving and disinfecting the knee area. With the knee in a flexed position, a sterile 23‐gauge syringe needle was inserted into the tibial marrow cavity via the intercondylar notch. After cutting off the excess needle hub, a mid‐diaphyseal closed fracture was created in the tibia using sterile surgical scissors.

After establishment of the injury models, a gelatin sponge loaded with 6 µL of the MSN‐CRISPRa_gRNA complex (containing 40 µg MSN‐PEI, 1.8 nmol *Cas9* mRNA, and 0.6 nmol gRNA per site) was placed into the injury area, followed by skin suturing. For the control group, a gelatin sponge loaded with an equal volume (6 µL) of PBS was implanted.

### 4.25 In Silico Off‐Target Prediction

Potential off‐target loci for the *Reln* gRNA sequence (5'‐TCCGGGCACAGCCCGAGGTG‐3') were predicted using the Cas‐OFFinder tool (http://www.rgenome.net/cas‐offinder/) against the mouse genome (Mus musculus, GRCm39), allowing up to two nucleotide mismatches.

### 4.26 Statistical Analysis

Statistical analysis was conducted with GraphPad Prism 7 (GraphPad, Inc., USA). Data were expressed as mean ± SD. Normality and homogeneity of variance were evaluated using the Shapiro‐Wilk test and Brown‐Forsythe test, respectively. Differences between two groups were evaluated using unpaired, two‐tailed Student's t‐tests. For comparisons of more than two groups, one‐way analysis of variance (ANOVA) followed by Bonferroni post‐hoc test was performed. For time‐course evaluations and multi‐factor experiments, two‐way ANOVA followed by Bonferroni's post‐hoc test was applied for multiple pairwise comparisons at indicated time points. The *P* values<0.05 were defined as statistically significant.

## Author Contributions

R.L Yang, C Xu, and J.H Jiang contributed to conception and design, data acquisition, analysis, and interpretation, and critically revised the manuscript; Yan Y contributed to nano particles synthesis and critically revised the manuscript; S Lin, Q.W Li, and D Liu contributed to experiment conduction, data analysis, and interpretation, critically revised the manuscript; M.Z Li contributed to vector design; H. Zhang contributed to sequencing data analysis. L.J Chen contributed to the *Reln^+/−^* mice maintenance. All authors gave final approval and agree to be accountable for the work.

## Conflicts of Interest

The authors declare no conflicts of interest.

## Supporting information




**Supporting File**: advs78530‐sup‐0001‐SuppMat.pdf.

**Supporting video 1**: advs78530‐sup‐0002‐VideoS1.mp4.

## Data Availability

The bulk RNA seq data is deposited in the China National GeneBank (CNGB) Sequence Archive (CNSA) under the accession code: PRJCA058228. The mass spectrometry data is deposited in the PRoteomics IDEntifications Database (PRIDE) under the accession code: PXD077621. Other data that support the findings of this study are available from the corresponding author upon reasonable request.
