# Supplementary material for: Reln mRNA Delivery Drives Neurogenic Bone Regeneration by Orchestrating Schwann Cell‐Mediated Exosomal Trafficking
Source: Adv Sci (Weinh). 2026 Nov 2:e78530. Online ahead of print. doi: 10.1002/advs.78530 (PMC13725739; doi:10.1002/advs.78530)
Supplement: Supplementary file 1 [file ADVS-9999-e78530-s002.pdf]

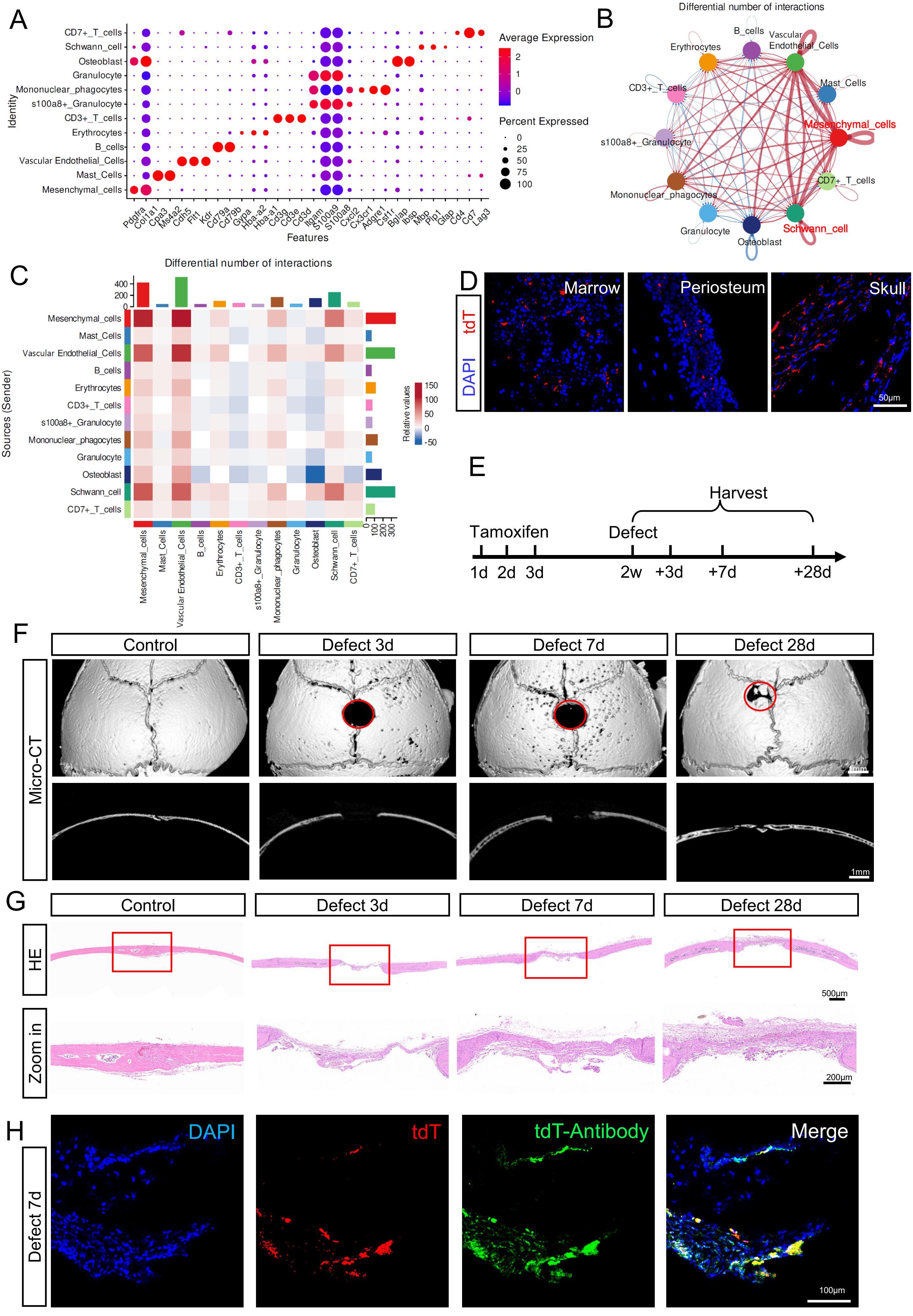

A
Average Expression
Percent Expressed
Identity
Features
B
Differential number of interactions
C
Differential number of interactions
Sources (Sender)
Relative values
D
Marrow
Periosteum
Skull
tdT
DAPI
50μm
E
Tamoxifen
Defect
Harvest
1d 2d 3d 2w +3d +7d +28d
F
Control
Defect 3d
Defect 7d
Defect 28d
Micro-CT
1mm
G
Control
Defect 3d
Defect 7d
Defect 28d
HE
Zoom in
500μm
200μm
H
DAPI
tdT
tdT-Antibody
Merge
Defect 7d
100μm

Supplementary Figure 1. Calvarial bone regeneration of *Plp-Cre^{ERT2}; Ai14* mice. (A) Dot plot showed the expression levels of key marker genes across the identified cell clusters. (B) Differential cell–cell interaction network showed changes in interaction numbers between groups. (C) Heatmap illustrated the differential number of interactions between all cell type pairs across the UNJ and INJ7 conditions. (D) The expression of tdTomato in bone marrow, periosteum, and skull from *Plp-Cre^{ERT2}; Ai14* mice. (E) Schematic diagram illustrated the experimental design. (F) 3D micro-CT reconstruction and representative 2D coronal view of mouse calvarial bone in *Plp-Cre^{ERT2}; Ai14* mice at 3-, 7-, and 28-days post-injury. (G) HE staining of mouse calvarial bone in *Plp-Cre^{ERT2}; Ai14* mice at 3-, 7-, and 28-days post-injury. (H) The expression of tdTomato and tdTomato-antibody in defect area from *Plp-Cre^{ERT2}; Ai14* mice at 7-day post-injury.

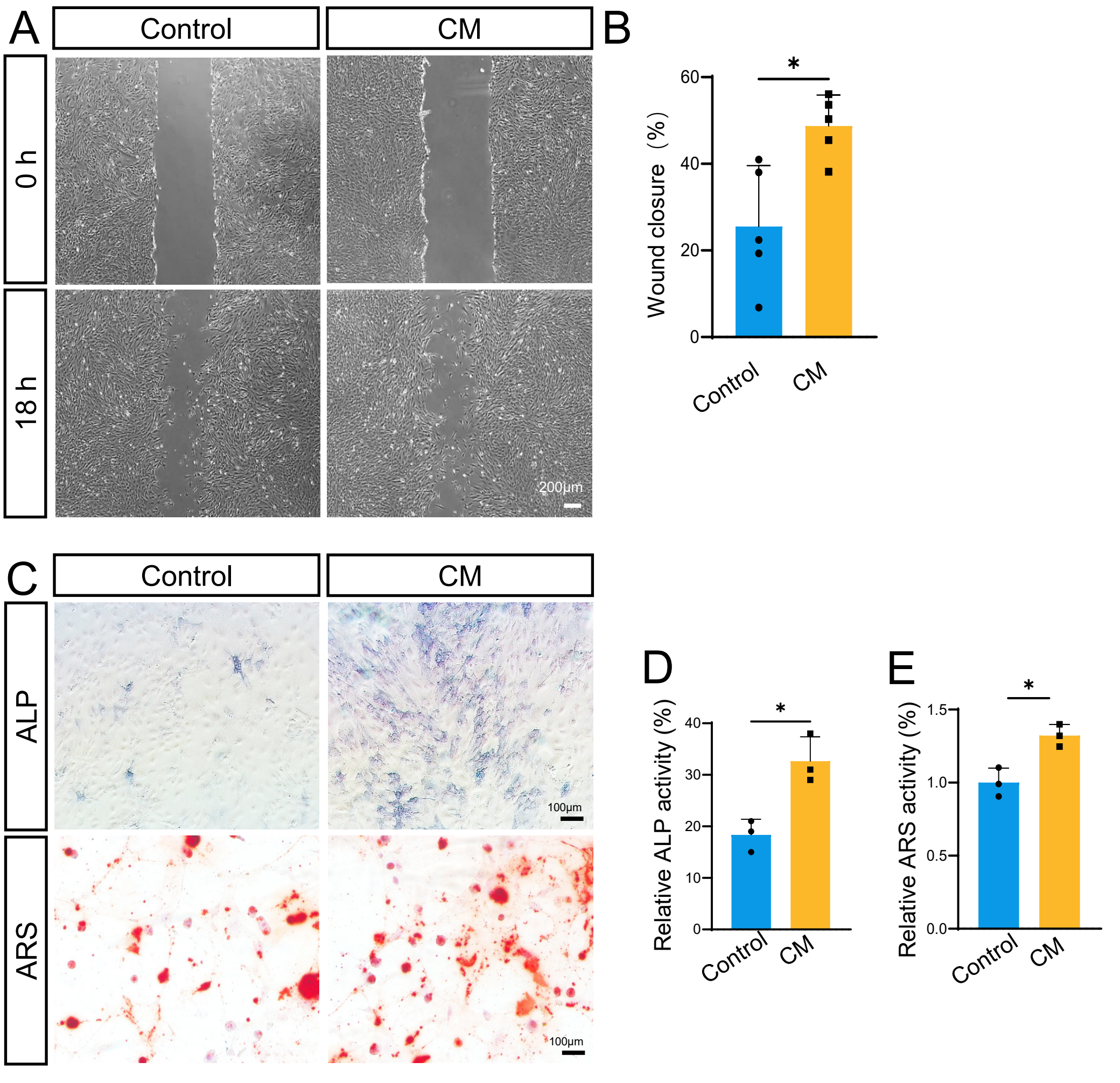

Supplementary Figure 2. Schwann cells promote osteogenesis of MSCs. (A) Representative images of the scratch area at 0 and 18h with or without Schwann cell-conditioned medium. (B) Quantification of scratch area in (A). (C) The ALP and ARS staining in control and Schwann cell-conditioned medium treated groups after osteogenic induction. (D, E) Quantification of ALP and ARS staining in (C). Each dot represents one individual replicate (n = 3 biological replicates in D, E; n = 5 biological replicates in B). Statistical significance was analyzed by unpaired Student's *t* test. Data are presented by Mean ± SD (*P < 0.05, **P < 0.01, ***P < 0.001).

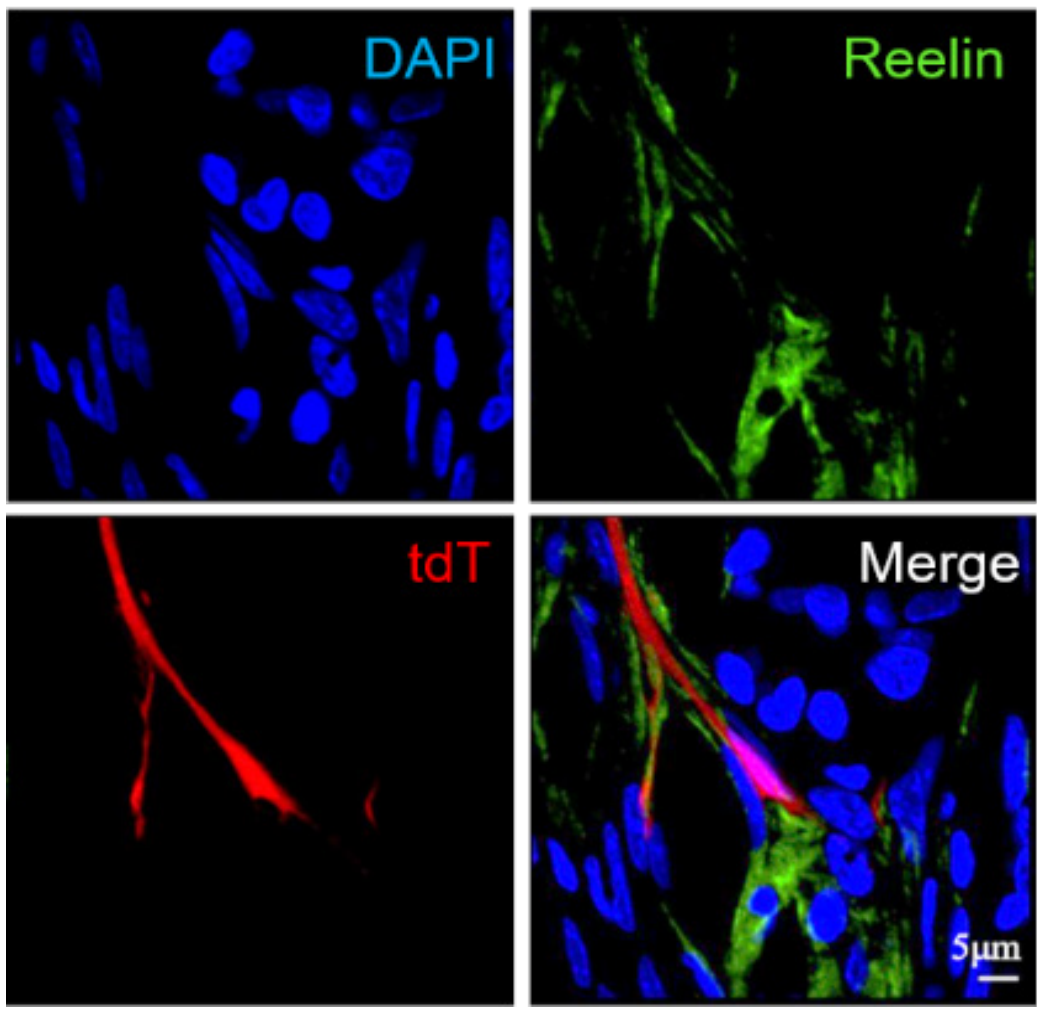

Supplementary Figure 3. The expression of Reelin (green) and Schwann cells (tdT) in the calvarial bone, presented by immunofluorescence staining from *Plp-CreERT2; Ai14* mice.

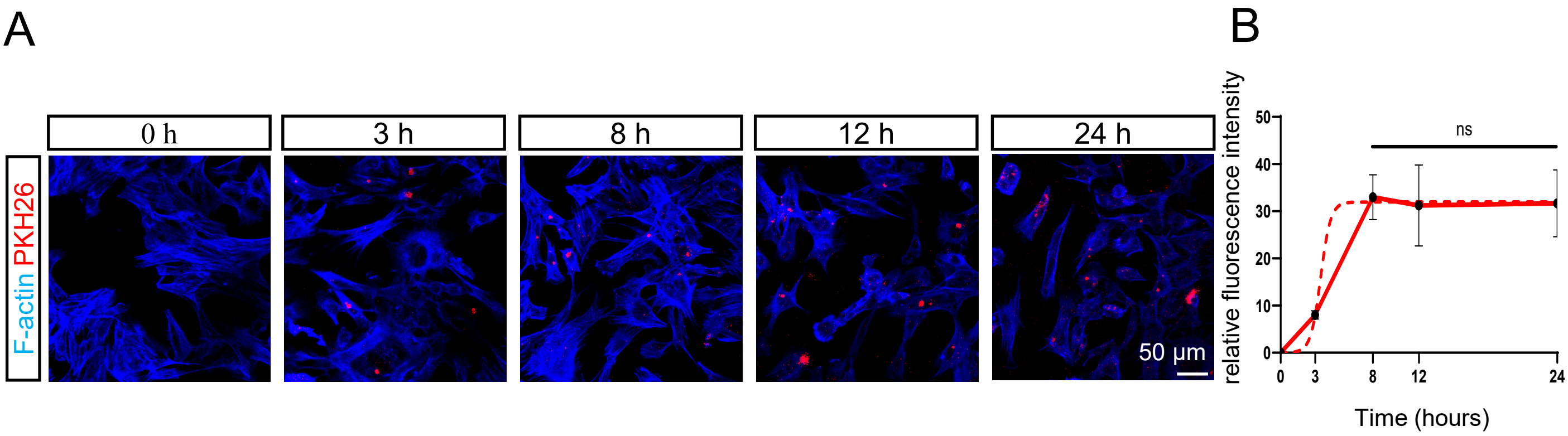

Supplementary Figure 4. The endocytosis of exosomes by MSCs is visualized using PKH26 labeling. (A) The MSCs internalize PKH26 labeled exosomes after treatment for 0, 3, 8, 12, 24 hours, presented by immunofluorescence staining. (B) Quantification of immunofluorescence intensity of PKH26 in (A). Data are presented by mean ± SD. Statistical significance was analyzed by One-way ANOVA with Bonferroni's post-hoc test

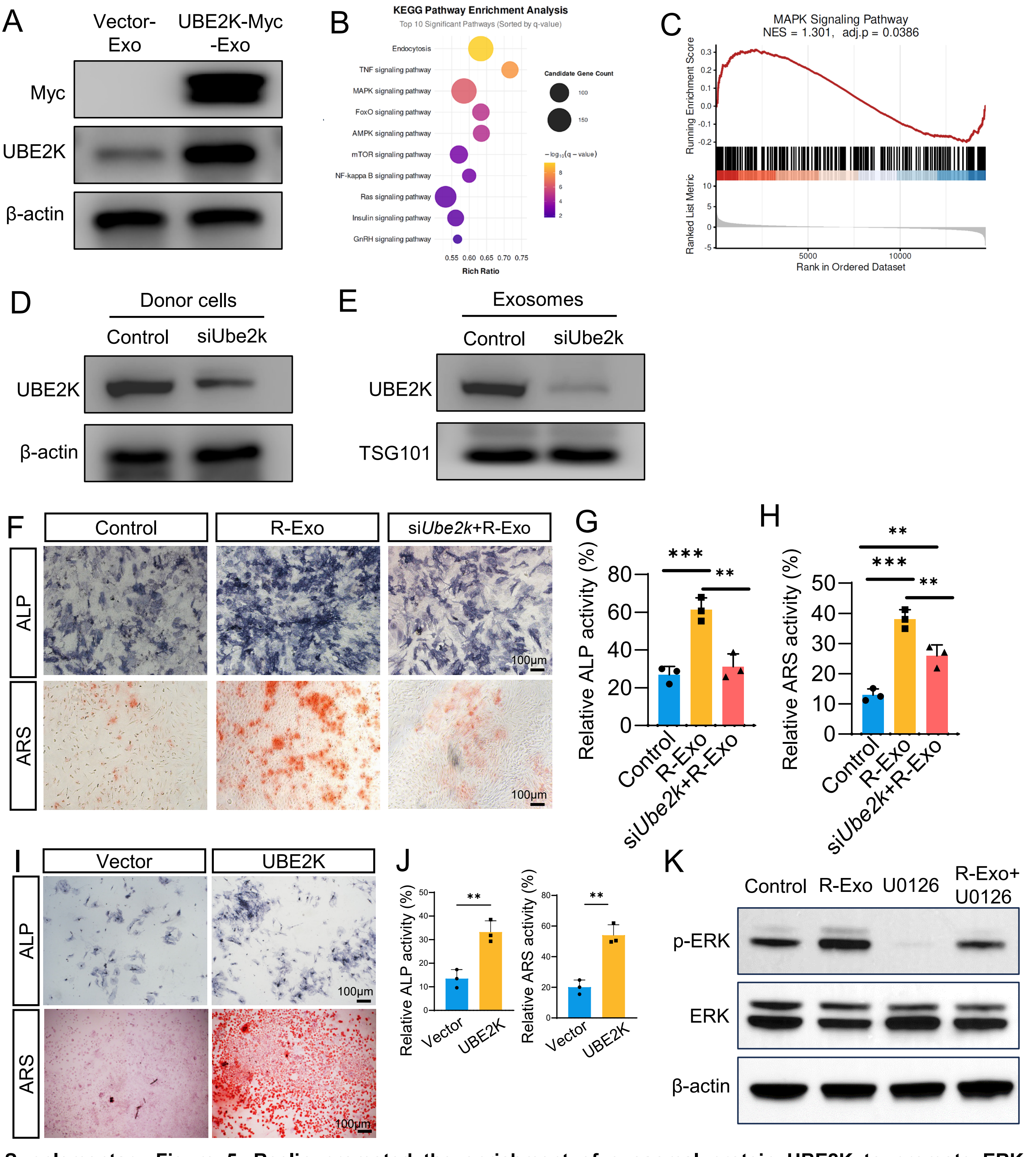

Supplementary Figure 5. Reelin promoted the enrichment of exosomal protein UBE2K to promote ERK activation.

(A) Western blot analysis of Myc and UBE2K expression in MSCs treated with exosomes derived from control or pCMV-UBE2K-Myc–transfected Schwann cells. (B) The KEGG enrichment pathways analysis of the upregulated DEGs in MSCs treated with exosomes derived from Reelin-stimulated Schwann cells. (C) GSEA enrichment plot of the MAPK signaling pathway in MSCs treated with Reelin-Exos. (D-E) Western blot analysis of UBE2K in Schwann and treated with or without pCMV-UBE2K-Myc. (F) The ALP and ARS staining in control, Reelin-stimulated exosomes, and Ube2k-knockdown Schwann cell-derived Reelin-stimulated exosomes groups. (G, H) Quantification of ALP and ARS staining in (F). (I) The ALP and ARS staining in the WT or UBE2K overexpression groups after osteogenic induction. (J) Quantification of ALP and ARS staining in (I). (K) Western blot analysis of phosphorylated ERK (p-ERK) and total ERK expression in MSCs treated with or without Reelin-stimulated exosomes or the ERK inhibitor U0126. Each dot represents one individual replicate (n = 3 biological replicates in G, H, and J). Statistical significance was analyzed by One-way ANOVA with Bonferroni's post-hoc test (G, H). Statistical significance was analyzed by unpaired Student's t test (J). Data are presented by Mean ± SD (**P* < 0.05, ***P* < 0.01, ****P* < 0.001).

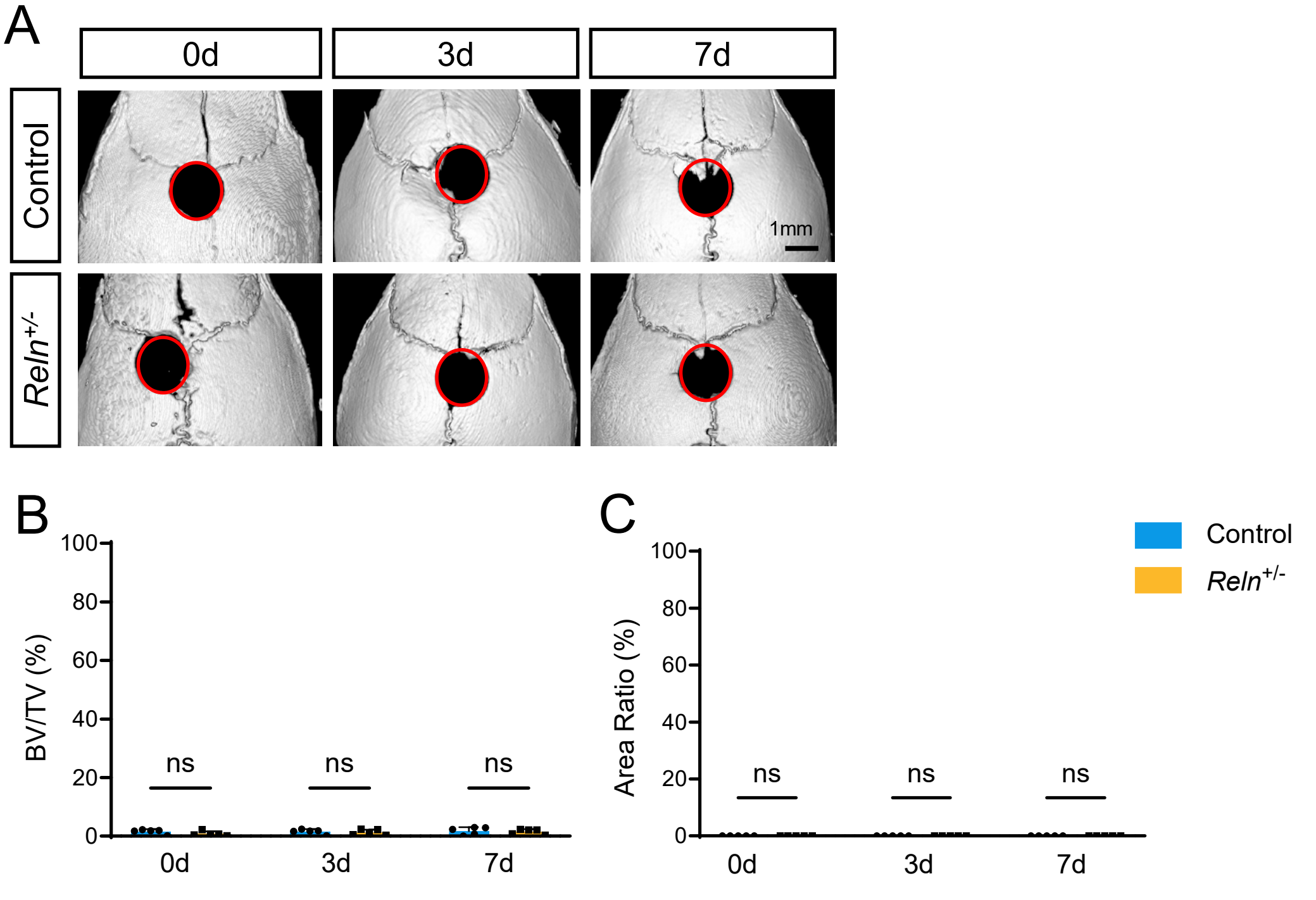

Supplementary Figure 6. (A) 3D micro-CT reconstruction of mouse calvarial bone in wild-type and *Reln*$^{+/-}$ mice at 0-, 3-, and 7-day post-injury. (B, C) Quantitative analysis of BV/TV and defect area ratio in regeneration area determined by micro-CT measurements at 0-, 3-, and 7-day post-injury. Each dot represents an individual mouse (n = 5 independent mice per group in B, C). Statistical significance was analyzed by Two-way ANOVA with Bonferroni post-hoc test (B, C). Data are presented by Mean ± SD.

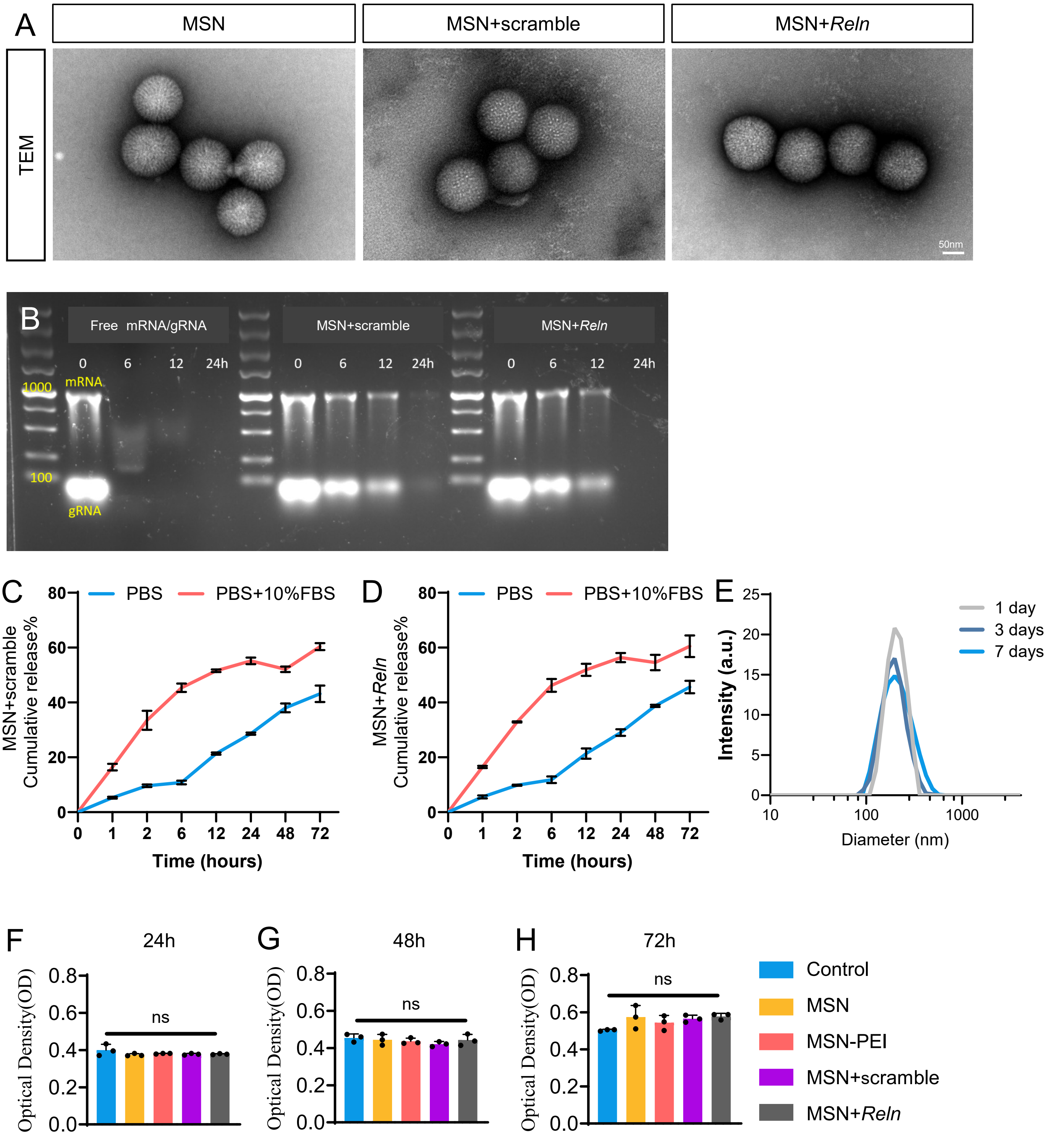

Supplementary Figure 7. Establishing Reln mRNA delivery system using MSN-PEI. (A) Representative Transmission Electron Microscopy (TEM) images of MSN (left) , MSN+scramble (middle) and MSN+*Reln* (right) after negative staining. (B) RNA retention of mRNA/gRNA from MSN+scramble and MSN+*Reln* was evaluated after incubation in DMEM supplemented with 10% FBS for 0, 6, 12, and 24 h. (C,D) RNA release assay from MSN+scramble and MSN+*Reln* was evaluated after incubation in PBS or PBS supplemented with 10% FBS for 0, 1, 2, 6, 12, 24, 48, and 72 h. (E) Dynamic light-scattering analysis of MSN+*Reln* after 1-, 3-, and 7-day. (F-H) CCK8 assay of Schwann cell after treatment with MSN, MSN-PEI, MSN+Scramble and MSN+*Reln* for 24, 48, and 72h. Data are presented by Mean ± SD of three independent experiments (C, D, F, G, and H). Statistical significance was analyzed by One-way ANOVA with Bonferroni's post-hoc test(F, G, and H).

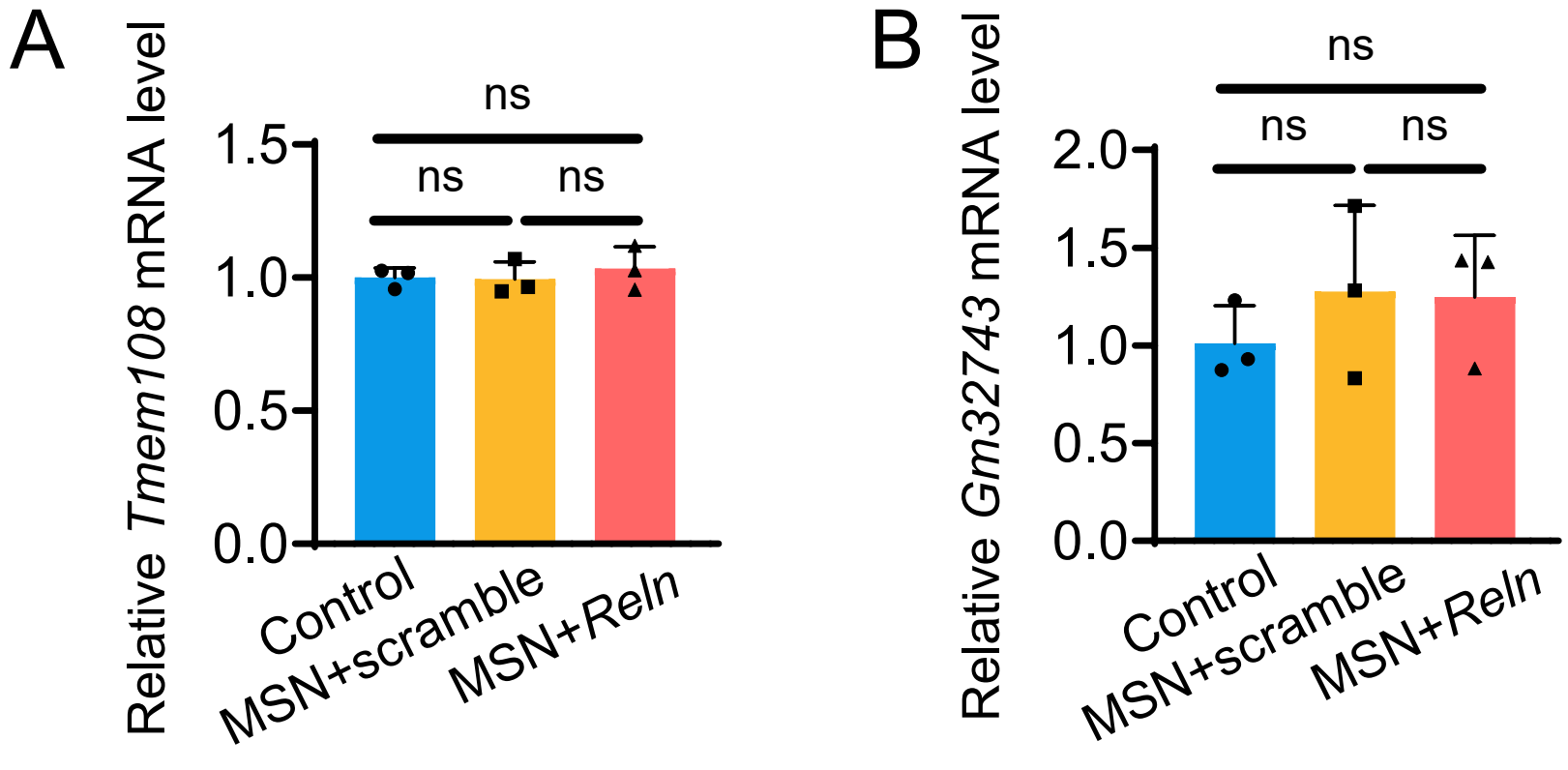

Supplementary Figure 8. Evaluation of predicted off-target gene expression. (A-B) The mRNA level of *Tmem108* (A) and *Gm32743* (B) in Control, MSN+Scramble and MSN+*Reln* groups, analyzed by RT-qPCR. Data are presented by Mean ± SD of three independent experiments

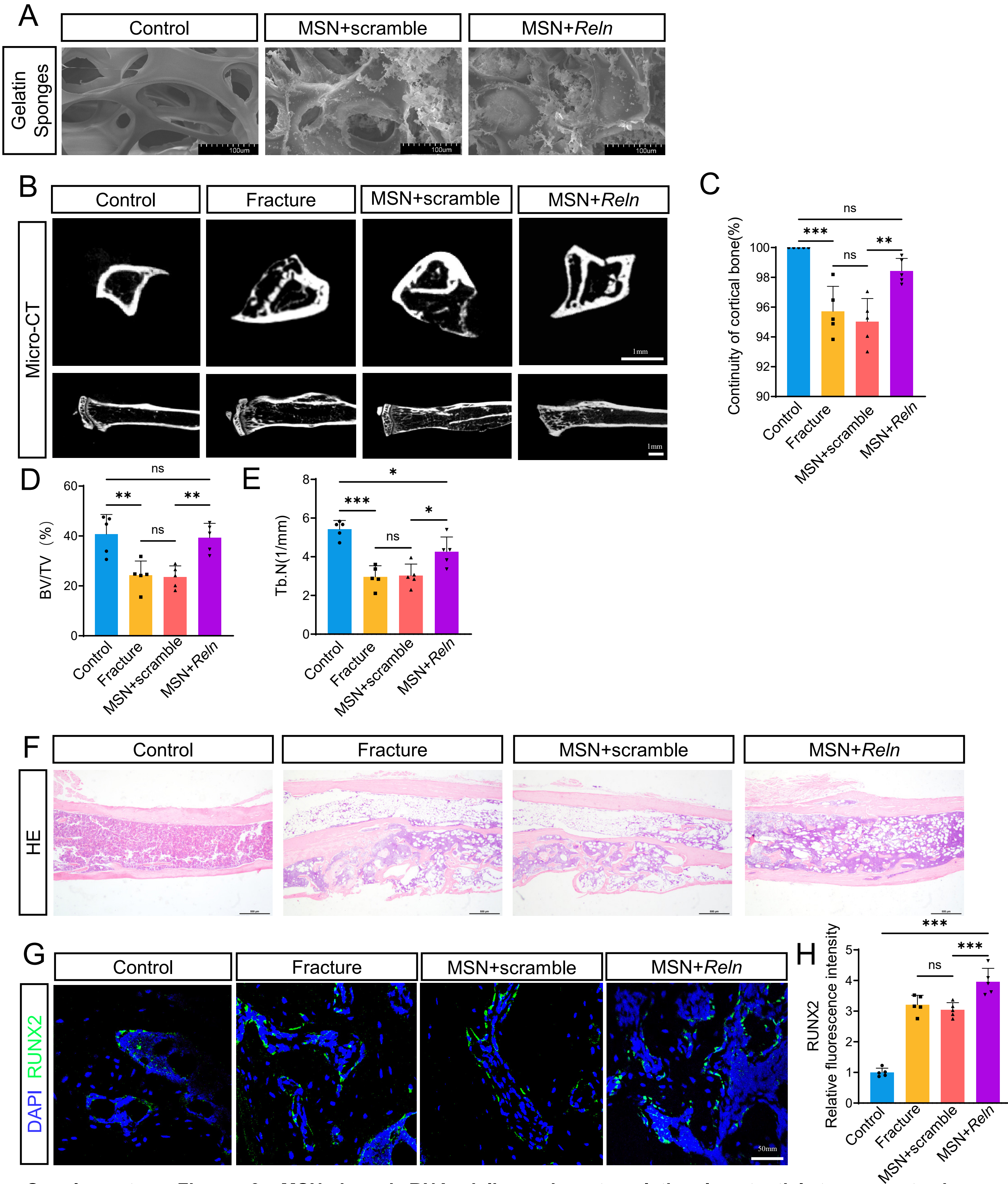

Supplementary Figure 9. MSNs-based RNA delivery has translational potential to promote bone regeneration. (A) Representative SEM images of gelatin sponges and gelatin sponges loaded MSN-CRISPRa_scramble and MSN-CRISPRa_Reln. (B) 3D micro-CT reconstruction and representative 2D coronal view of mouse tibia in control and fracture model with or without CRISPRa_Reln-loaded MSN treatment at 4 weeks post-injury. (C-E) Quantitative analysis of continuity, BV/TV, and Tb. N in fracture area determined by micro-CT measurements at 4 weeks post-injury. (F) HE staining of tibia in control and fracture model with or without CRISPRa_*Reln*-loaded MSN treatment at 4 weeks post-injury. (G) The expression of RUNX2 in tibia with or without CRISPRa_*Reln*-loaded MSN treatment at 4 weeks post-injury, presented by immunofluorescence staining from WT mice. (H) Quantitative analysis of RUNX2 intensity in (G). Each dot represents an individual mouse (n = 5 independent mice per group in C, D, E, H). Statistical significance was analyzed by One-way ANOVA with Bonferroni's post-hoc test(C, D, E, H). Data are presented by Mean ± SD of five independent experiments (*P < 0.05, **P < 0.01, ***P < 0.001).

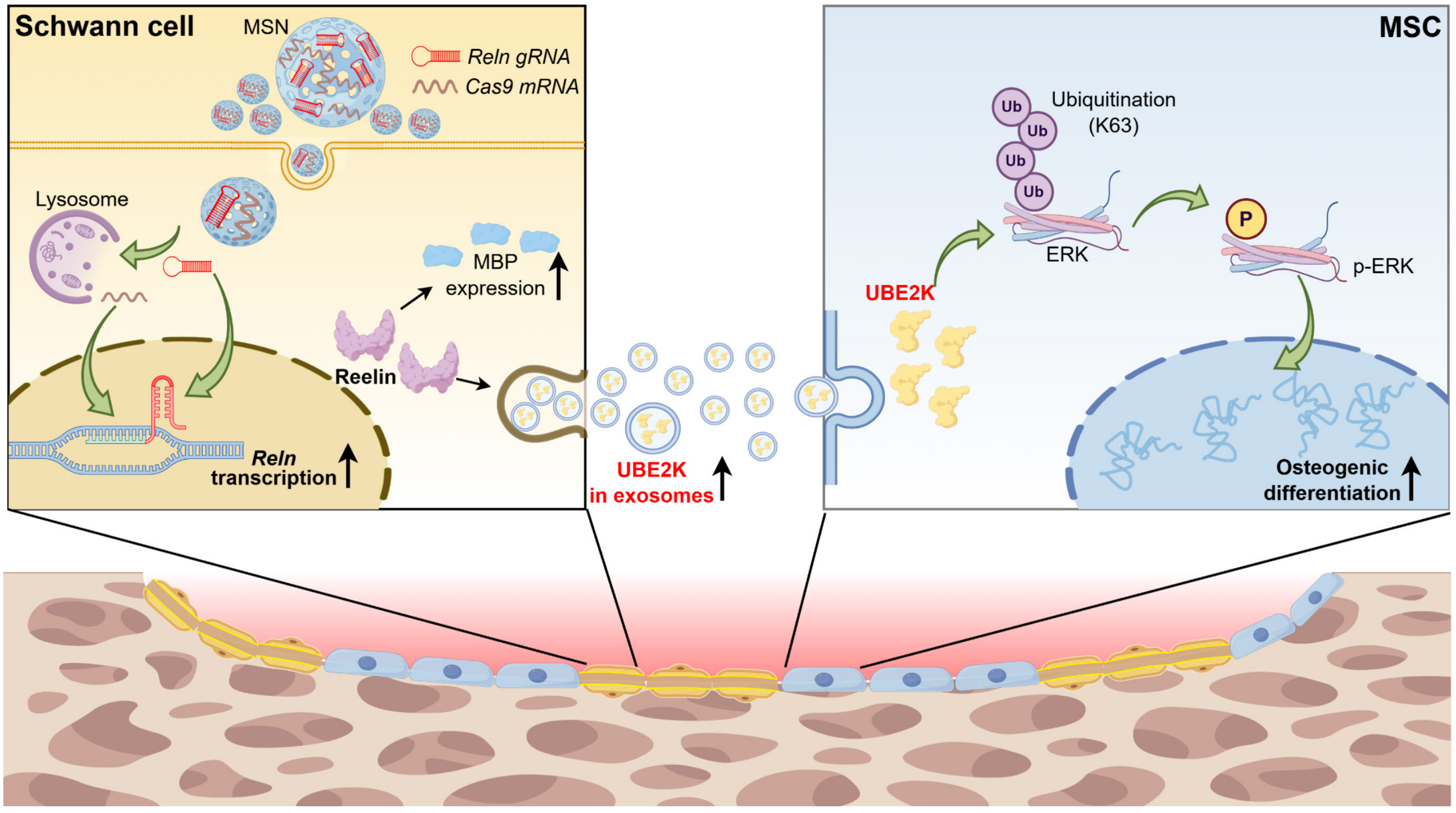

Supplementary Figure 10. Schema. MSNs serve as a robust vehicle for CRISPRa-mediated *Reln* induction, driving bone repair through the establishment of a neurogenic-supportive microenvironment. The schema was drawn by Figdraw.

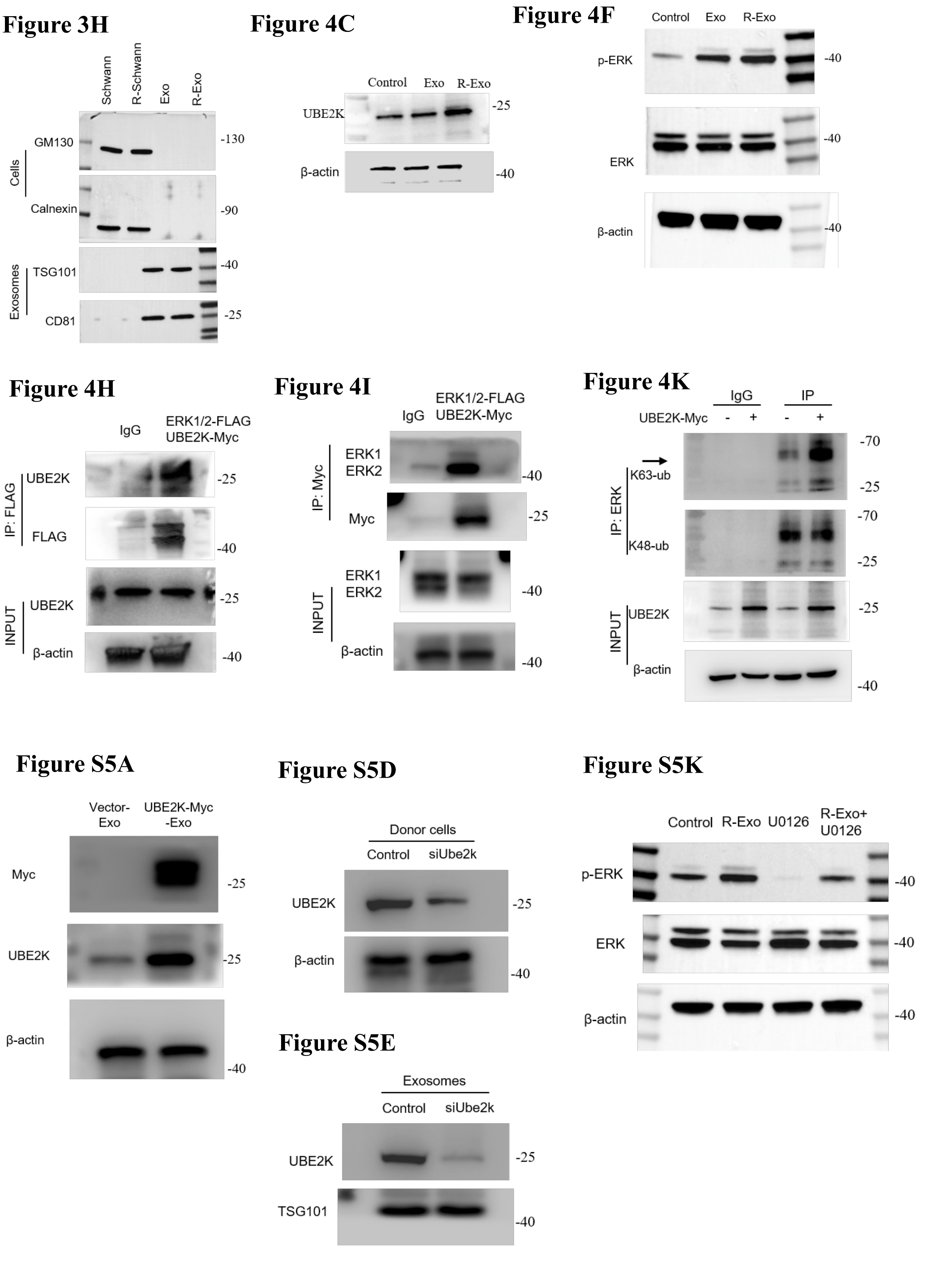

Supplementary Figure 11. Uncropped western blot images.

Table S1. qRT-PCR mouse primer sequences

Gene	F/R	Primer sequence
Gapdh	F	GGTTGTCTCCTGCGACTTCA
	R	TGGTCCAGGGTTTCTTACTCC
Reln	F	GTCATTCCTGGAGCCACAGATC
	R	GGACATCATCCAAAGCCCACTG
Tmem108	F	CCCACCTCTAGCTGTGCC
	R	TCATACTGGCTCCCAGAG
Gm32743	F	TCCGGGCACAGCTAGAGG
	R	GCTCTGTCACACGTGGTC
